# Constant-pH Simulations with the Polarizable Atomic Multipole AMOEBA Force Field

**DOI:** 10.1021/acs.jctc.3c01180

**Published:** 2024-03-20

**Authors:** Andrew C. Thiel, Matthew J. Speranza, Sanika Jadhav, Lewis L. Stevens, Daniel K. Unruh, Pengyu Ren, Jay W. Ponder, Jana Shen, Michael J. Schnieders

**Affiliations:** † Department of Biomedical Engineering, University of Iowa, Iowa City, Iowa 52242, United States; ‡ Department of Biochemistry, University of Iowa, Iowa City, Iowa 52242, United States; § Department of Pharmaceutical Sciences and Experimental Therapeutics, University of Iowa, Iowa City, Iowa 52242, United States; ∥ Office of the Vice President for Research, University of Iowa, Iowa City, Iowa 52242, United States; ⊥ Department of Biomedical Engineering, University of Texas, Austin, Texas 78712, United States; # Department of Chemistry, Washington University in St. Louis, St. Louis, Missouri 63130, United States; ∇ Department of Pharmaceutical Sciences, University of Maryland School of Pharmacy, Baltimore, Maryland 21201, United States

## Abstract

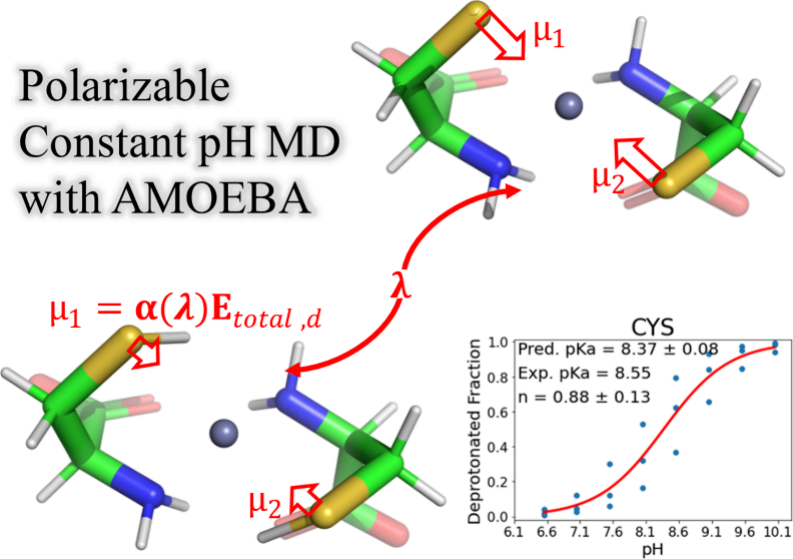

Accurately predicting protein behavior across diverse pH environments remains a significant challenge in biomolecular simulations. Existing constant-pH molecular dynamics (CpHMD) algorithms are limited to fixed-charge force fields, hindering their application to biomolecular systems described by permanent atomic multipoles or induced dipoles. This work overcomes these limitations by introducing the first polarizable CpHMD algorithm in the context of the Atomic Multipole Optimized Energetics for Biomolecular Applications (AMOEBA) force field. Additionally, our implementation in the open-source Force Field X (FFX) software has the unique ability to handle titration state changes for crystalline systems including flexible support for all 230 space groups. The evaluation of constant-pH molecular dynamics (CpHMD) with the AMOEBA force field was performed on 11 crystalline peptide systems that span the titrating amino acids (Asp, Glu, His, Lys, and Cys). Titration states were correctly predicted for 15 out of the 16 amino acids present in the 11 systems, including for the coordination of Zn^2+^ by cysteines. The lone exception was for a HIS-ALA peptide where CpHMD predicted both neutral histidine tautomers to be equally populated, whereas the experimental model did not consider multiple conformers and diffraction data are unavailable for rerefinement. This work demonstrates the promise polarizable CpHMD simulations for p*K*_a_ predictions, the study of biochemical mechanisms such as the catalytic triad of proteases, and for improved protein–ligand binding affinity accuracy in the context of pharmaceutical lead optimization.

## Introduction

A ubiquitous regulator of protein structure is pH, which is exemplified by the impact of low pH on the stability and folding of proteins,^1^ even causing proteins to denature.^2^ Misregulated pH can contribute to detrimental effects such as the formation of amyloid fibrils in Alzheimer’s disease^3^ and insulin aggregation.^4^ These effects on structure propagate to protein functions that drive diverse activities such as structural dynamics,^5^ ligand binding,^6^ and enzyme activity.^7^ For example, the M2 protein from the influenza A virus is a pH-activated proton channel that mediates the acidification of the interior of viral particles entrapped in endosomes.^8^ Other examples include the regulation of water uptake via aquaporin gating by cytosolic pH in plant root cells^9^ and a low-pH induced conformational change in hemagglutinin that activates fusion activity in the influenza virus.^10^ Understanding these effects in ligand binding is also critical to drug design where approximately 78% of oral drugs have varying protonation states.^11^ The electrostatic contribution to these processes cannot be fully described without accurate knowledge of all fluctuating protonation states, especially considering that for an average protein about 25% of residues have titratable protons and that charge–charge interactions produce some of the strongest atomic forces at work in proteins.

There exist many strategies for the calculation of protein titration curves and residue p*K*_a_ values. These techniques vary in both complexity and speed, from rigorous quantum mechanics^12,13^ to empirical rule-based heuristics.^14−17^ Even among those methods grounded in *ab initio* chemical theory there is a great deal of variety with respect to the treatment of dielectric screening, the calculation of electrostatic contributions, polarization effects, titration coupling, and conformational sampling. Constant-pH molecular dynamics extends past mere p*K*_a_ prediction, offering an understanding of titration-coupled conformational dynamics through which many protein functions act.^18^

## Foundations of Constant-pH Molecular Dynamics

The foundations for most contemporary advanced titration models were described in 1997 by Baptista et al.^19^ Recognizing the sensitivity of protonation energies to small changes in atomic coordinates, they devised a method by which protein titration could be observed as a function of these movements (i.e., during the course of a molecular dynamics simulation). Their technique, known as constant-pH molecular dynamics (CpHMD), combines molecular mechanics with continuum electrostatic pH calculations by adding the charge state at each titratable site as a parameter in the MD simulation. Fractional protonation states are made available to each site and are updated after a defined number of MD steps via Poisson–Boltzmann (PB) calculations.

Five years later, Baptista et al.^20^ modified their CpHMD method to affect a departure from unphysical fractional protons. Rather than allow continuously variable charge on titratable residues, their solution employed a Metropolis Monte Carlo^21^ (MC) criterion at regular intervals during simulation to switch directly and instantaneously between fully protonated and fully deprotonated states. The PB equation was used in evaluating the MC moves between the titration states. Mongan, Case, and McCammon employed the generalized Born (GB) equation in their CpHMD methods to improve the sampling speed over PB methods.^22^ Bürgi and colleagues introduced a variation^23^ on Baptista’s theme where a costly explicit-solvent thermodynamic free energy simulation was used to estimate the free energy of deprotonation for each MC move. A significant acceleration to sampling discrete protonation states was achieved by Roitberg and colleagues through their implementation of replica exchange on hybrid MD/MC simulations.^24,25^ Although nonphysical intermediate states are not sampled using discrete approaches, much of the recent work in this area has continued to use a continuous approach, as it can easily be applied to all-atom simulations and it is efficient. However, nonequilibrium MD/MC approaches are being explored to overcome the issue of the protonation state flip being rejected in MC.^26,27^ One continuous approach pioneered by Brooks and co-workers^28,29^ was the application of λ-dynamics to update titration states. In this approach, a λ-coordinate corresponding to each titration state is added to create an extended Hamiltonian. This λ-coordinate is propagated according to the equations of motion governed by the potential of its surroundings and the environmental pH. The λ-dynamics on titratable sites approach has become more widely adopted and can now be found within the GROMACS,^30^ CHARMM,^31,32^ and Amber^33,34^ MD packages. Continuous approaches have been expanded to optionally include long-range electrostatics via particle-mesh Ewald (PME) summation by Shen et al.^32,35^ and titratable waters to maintain zero net charge for PME.^36^

## Efficient Alchemical Paths in a Polarizable Multipole Context

At present, the state-of-the-art implementation^37^ of arbitrary thermodynamic path sampling under a polarizable multipole potential is to compute the electrostatic energy of a dual topology configuration via linear interpolation between end states. The self-consistent fields (SCFs) for each topology are converged separately prior to interpolation. Since convergence of the SCF is a bottleneck in the evaluation of the energy and forces for a polarizable multipole model, the need to compute two SCFs makes dual topology calculations relatively less attractive. Here we describe, for the first time, an analytical thermodynamic path for AMOEBA that requires only a single SCF evaluation per energy and gradient evaluation.

## Theory and Implementation

### Continuous CpHMD for the Polarizable AMOEBA Model with PME

The current implementation supports the residues ASP, GLU, HIS, LYS, and CYS. Following a common approach established by fixed partial charge CpHMD models, the protonation states of titratable residues are propagated alongside the atomic Cartesian coordinates during dynamics. This is described by an extended Hamiltonian laid out by Shen and co-workers,^32^
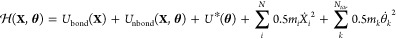
1where *i* is the index over atoms and *k* is the index over both titration and tautomer extended system variables, **X** is the Cartesian coordinate vector, and **θ** is a vector of both titration and tautomer extended system states. The titration (*λ*_*k*_) and tautomer (*ζ*_*k*_) states are bound between 0 and 1 through the relation *λ*_*k*_or *ζ*_*k*_ = sin^2^(*θ*_*k*_). The first term on the right-hand side of eq 1 is the potential due to all bonded terms. The bonded terms were not scaled with titration states such that a deprotonated atomic site retains bonded terms with an alchemically decoupled proton.^29^ The second term is the nonbonded potential, which is a function of both Cartesian and titration/tautomer coordinates. The nonbonded energy terms and their derivatives are described in the next section. The third term is the biasing potential, which is a function of only the titration and tautomer states. The bias is composed of three components
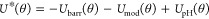
2

The first component represents a barrier centered at 0.5 that suppresses the intermediate titration and tautomer states.
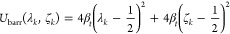
3

This barrier also affects the kinetics of the transition between end states by specifying the magnitude of the barrier (*β*_*t*_). The ζ-dependent term is not present for LYS and CYS residues that lack tautomers. The model potential, *U*_mod_(**θ**), is a potential of mean force (PMF) for protonation of the model compound
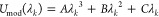
4

This PMF flattens the free-energy surface between the protonated and deprotonated states such that the model compound’s titration state is dictated by its environment of nonbonded interactions and the final bias term. The PMF shown in eq 4 applies to all residues where there is no tautomerism (the PMF functional form for other cases is given in the Supporting Information). The symmetry of ASP and GLU oxygen atoms implies that their PMF will show no energy difference between end states. In the case of HIS, three PMFs were collected. Two for the protonation between the distinct tautomers, HIE and HID, and one between the two tautomer states (see Simulation Protocol). The pH bias, *U*_pH_(**θ**) is the final term and represents the relative free energy of deprotonation at the environmental pH
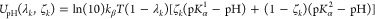
5where *k*_*β*_ and *T* are the Boltzmann constant and temperature, respectively. The scaling by (1 – *λ*_*k*_) is consistent with the convention used here whereby *λ*_*k*_ = 1 denotes a protonated site. As before, when LYS and CYS are considered, there is no ζ-dependence.

### van der Waals Interactions

Under the AMOEBA model,^38−40^ a buffered 14-7 van der Waals^41^ term is used, which can be modified to include soft-core capabilities that allow for smooth decoupling as λ goes to zero. The soft-core form between atoms *i* and *j* is given by
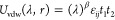
6where *t*_1_ and *t*_2_ are defined as
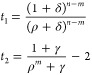
7and the buffered 14-7 constants are
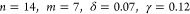


In the above expressions, *ε*_*ij*_ is the well depth and ρ is the normalized atomic separation distance (*r*_*ij*_/*r*_min,*ij*_) defined by the ratio of the minimum-energy separation distance *r*_min,*ij*_ and the current separation distance *r*_*ij*_.

In the case of an alchemical proton, preliminary work demonstrated that the soft-core term is not necessary because each proton is protected by the van der Waals repulsion provided by its heavy atom. For this reason, the extended system van der Waals functional form given by
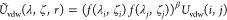
8is simply a scaled version of the default (nonsoft core) van der Waals energy, where the scaling is controlled by titration and tautomer variables. These scaling contributions are separable and are defined for each residue in Table 1.

**Table 1:** Scaling Contributions for the van der Waals Energy Are Given in Equation 8^a^

Proton	Nontitrating	LYS, CYS	ASP, GLU	HIS
*f*(λ, ζ)	1	*λ*_*i*_	*λ*_*i*_*f*^*m*^ (*ζ*_*i*_)	((1 – *λ*_*i*_) *f*^*m*^ (*ζ*_*i*_) + *λ*_*i*_)

a Here  represents the tautomer state and is based on the tautomer direction that is assigned at initialization to the titrating hydrogens. For histidine, the HE2 proton is assigned as 1 and the HD1 proton is assigned as −1.

### Permanent Atomic Multipoles as a Function of Titration and Tautomer States

The permanent atomic multipole for atom *i* is described by a vector of multipole coefficients
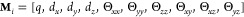
9

If the multipole is a function of only a titration state variable *λ*_*k*_ as is the case with lysine, then **M**_*i*_(*λ*_*k*_) is given by a linear function of the permanent atomic moments for the unprotonated **M**_*i*_^(U)^ and protonated **M**_*i*_^(P)^ states
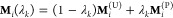
10and the partial derivative of **M**_*i*_(*λ*_*i*_) with respect to *λ*_*k*_ is
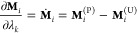
11

If the multipole is also a function of the tautomer state variable *ζ*_*k*_, then **M**_*i*_(*λ*_*k*_, *ζ*_*k*_) is given by a mixing of linear functions between permanent moments for the unprotonated and protonated states and between tautomeric moments. In the case of protonated tautomerism, there is mixing between the unprotonated moment and two protonated moments **M**_*i*_^(P1)^ and **M**_*i*_^(P2)^
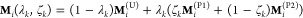
12and the partial derivative of **M**_*i*_(*λ*_*k*_, *ζ*_*k*_) with respect to *λ*_*k*_ is
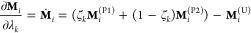
13and the partial derivative with respect to *ζ*_*k*_ is
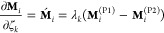
14

The rotation of multipole moments from their local chemical frame into the global frame is performed for both **M**_*i*_ and its partial derivatives **Ṁ**_*i*_ and **Ḿ**_*i*_ after the linear functions defined above have been evaluated. For this reason, both the local coordinate frame convention (e.g., Z-then-X or Z-then-bisector) and its frame-defining atoms must be identical for both unprotonated and protonated end states.

### Real Space Permanent Multipole Energy and Partial Derivatives

To describe the permanent multipole energy, we introduce multipolar operators^42,43^
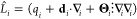
15
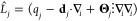
16

The partial derivative of *L̂*_*i*_ or *L̂*_*j*_ with respect to *λ*_*k*_ is achieved by substituting the rotated **Ṁ**_*i*_ or **Ṁ**_*j*_ multipole moments into eq 15 or eq 16, respectively, to give
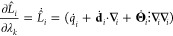
17
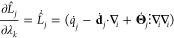
18

The partial derivative of *L̂*_*i*_ or *L̂*_*j*_ with respect to *ζ*_*k*_ is the same as the above but with rotated **Ḿ**_*i*_ or **Ḿ**_*j*_ from eq 14 substituted instead.

Let **n** be a vector of integer triples such that **n** = *n*_1_**a** + *n*_2_**b** + *n*_3_**c**, where {**a**, **b**, **c**} are the unit cell vectors along the *a*, *b*, and *c* axes. Then summing over **n** accomplishes the interaction with periodic images. For the case of **n** = {0, 0, 0}, *i* = *j* is omitted (i.e., self-interactions within the central unit cell, which is denoted by the prime on **n**′ below).
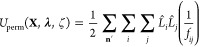
19where *f*_*ij*_ = *r*_*ij*_ + α(1 – *λ*_*k*_*λ*_l_)^2^ augments *r*_*ij*_ = |**r**_*i*_ – **r**_*j*_ + **n**| with soft-core support.

Using the Ewald range separation approach, the short-range real space interaction potential is given by
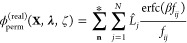
20where β is the tunable Ewald parameter. The real space permanent multipole energy is then given by
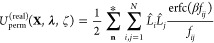
21

Differentiating the real space permanent energy with respect to *λ*_*k*_ gives
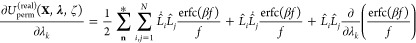
22

The derivative of the real space permanent energy with respect to *ζ*_*k*_ is the same as above but uses  and . Titrating hydrogens can be treated with soft-core electrostatic interactions to protect their charge site at small values of λ when their van der Waals interactions are not present. However, the titrating hydrogen atoms are not treated with soft-core electrostatics for the same reason described above for neglecting a soft core for the van der Waals term. In this case, the third term of eq 22 is eliminated because *f* is not a function of λ.

### Reciprocal Space Permanent Multipole Energy and Partial Derivatives

From Sagui et al., the reciprocal space multipolar electrostatic energy *U*_rec_ is given by^42^
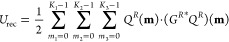
23where *Q*^*R*^ is the reciprocal lattice grid populated with the splined multipoles (*θ*_*p*_)
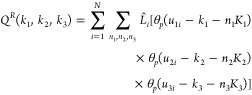
24

And *G*^*R*^ is the discrete Fourier transform of the coefficients arising from the structure factor. The partial derivative of eq 23 with respect to *λ*_*k*_ is given as
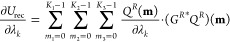
25

In other words, the splined **M**_*i*_ (eq 12) is the reciprocal space source term, and its reciprocal space potential is felt by ∂**M**_*i*_/(∂λ_*k*_) = **Ṁ**_*i*_ or ∂**M**_*i*_/ζ_*k*_ = **Ḿ**_*i*_ (eq 13, eq 14). This is accomplished by computing the reciprocal space potential using **M**_*i*_ and then evaluating the partial derivatives using **Ṁ**_*i*_ or **Ḿ**_*i*_.

### Polarizability as a Function of Titration and Tautomer States

The AMOEBA polarization model is based on isotropic polarizabilities and, in general, a single polarizability value per element (i.e., all carbons share the same polarizability value). Polarizabilities that change as a function of a titration state (*λ*_*k*_) and tautomer state (*ζ*_*k*_) can be defined as **α**_*i*_(*λ*_*k*_, *ζ*_*k*_) using a linear function between unprotonated **α**_*i*_^(U)^ and protonated **α**_*i*_^(P)^ states and a linear function between tautomer states. This is analogous to the functions used to interpolate atomic multipole coefficients. Equation 26 is an example of the case of protonated tautomerism
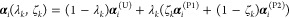
26

For most heavy atoms in the AMOEBA protein force field, polarizability is independent of titration state or tautomer state, while for titrating hydrogen, the expression reduces to linearly turning the polarizability on (or off). The exception to this rule is for sulfur and carboxylic oxygen atoms, such that interpolation occurs between two nonzero polarizabilities. A complication that arises from interpolating between two nonzero values is in the Thole damping model, where the charge density (ρ) takes the form
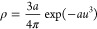
27where *u* = *R*_*ij*_/(*α*_*i*_*α*_*j*_)^1/6^ is the effective separation as a function of the two polarizabilities at sites *i* and *j*. As the polarizability is a function of state variables (*λ*_*k*_, ζ_*k*_), so too must be the charge density. However, an approximation made by this model is to fix the charge density such that there is no derivative of the charge density with respect to either *λ*_*k*_ or ζ_*k*_. Therefore, titrating sulfur and carboxylic oxygen atom charge densities are fixed to a value as calculated by the average of their end-state polarizabilities. This is not necessary for titrating hydrogen atoms where their polarizability will be zero at one end state and will not contribute in any interaction.

### Polarization Energy and Partial Derivatives

The following derivation will be presented in the context of a vacuum calculation for simplification, such that the field contributions exclude reciprocal space and self-correction terms. However, the derivatives of these terms will be given following real space derivation. The polarization energy is given by an inner product
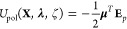
28where **μ** and **E**_*p*_ are vectors of length 3*n* that contain the induced dipoles and permanent multipole field components, respectively. The subscript *p* indicates that 1–2, 1–3, etc. bonded masking rules are applied in calculating the field at each induced dipole site. For the AMOEBA model, the induced dipole at site *i* is a function of an isotropic polarizability α_*i*_ interacting with the total field^39^
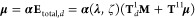
29

Here, **M** is a vector of length 13*n* that contains multipole moments. Given the definition of **T**_*ij*_
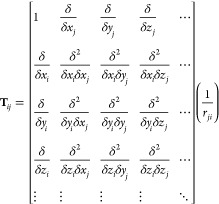
30as the multipole–multipole interaction matrix between sites *i* and *j*, ***T***_*ij*_^1^ is the 3 × 13 interaction matrix corresponding to the second through fourth rows of **T**_*ij*_. In the condensed phase under periodic boundary conditions, **T**_*ij*_ will use erfc (*βf*_*ij*_)/*f*_*ij*_ in place of 1/***r***_*ji*_. ***T***_*d*_^1^ is a super matrix with elements of tensor ***T***_*ij*_^1^ (varying *i* and *j*). Subscript *d* indicates the use of the AMOEBA group-based polarization masking rules used to compute the direct field due to permanent atomic multipoles. In the same vein, **T**^11^ is a supermatrix with elements corresponding to the tensor ***T***_*ij*_^11^, which is a submatrix of ***T***_*ij*_^1^ that contains only the dipole–dipole interaction terms and in condensed phase would include the Thole damping modification to elements of the matrix. We solve for the induced dipoles and then factor out **μ**:
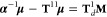
31
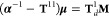
32

For convenience, a quantity **C** is defined and substituted above to simplify
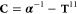
33
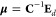
34where **E**_*d*_ = **T**_*d*_^1^**M** is the permanent field using polarization group masking rules. The partial derivative of the polarization energy with respect to an atomic coordinate is then given by the product rule as
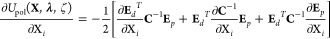
35

Additional simplification is achieved through the use of the following two relationships:
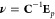
36
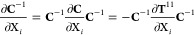
37

This gives the partial derivative of polarization energy with respect to an atomic coordinate of site *i*:
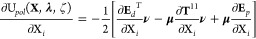
38

The derivation with respect to an extended system variable proceeds in a similar fashion:
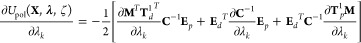
39

Using the expression for the lambda derivative of the quantity **C** gives
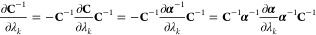
40

Substituting into the central term on the right-hand side of eq 39 gives
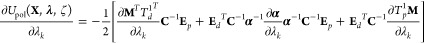
41

Rearrangement of eq 29 using eq 34 reveals the following relationships
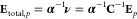
42
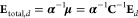
43

This allows the simplification of eq 41 to
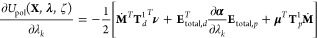
44

The first and third terms are equivalent in form to the computation of a polarization energy, as defined in eq 28, but using partial derivatives of the permanent multipole moments with respect to a titration or tautomer variable. The central term is trivial to compute because the 3*n* × 3*n* matrix **α** is diagonal and the only elements of ∂**α**/(∂λ_*k*_) that are nonzero arise from polarizabilities that are a function of λ_*k*_ (e.g., the titrating hydrogen, sulfur, or carboxylic oxygen atoms of a single residue).

In eq 44, the total field is due to permanent multipoles and induced dipoles. The permanent field can be broken down further into real, reciprocal, and self-contributions
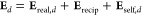
45
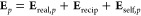
46

While the middle term on the right-hand side of eq 44 explicitly uses the total field, the first and last terms use the permanent multipole field. These terms resemble the interaction of the titrating multipoles with the field of the induced dipoles. Now considering these terms in the condensed phase with a reciprocal space component and reorganizing eq 44, we can simply define the derivatives as
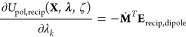
47where **E**_recip,dipole_ = (**E**_recip,ν_ + **E**_recip,μ_)/**2**. The same logic can be followed for the self-correction term,
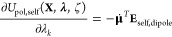
48where .

### Modification of AMOEBA Force Field Parameters

As discussed previously,^40^ dipoles and quadrupoles are defined in a local frame based on the coordinates of neighboring atoms. A crucial element for the interpolated multipoles is that their frames and frame-defining atoms must be consistent between the end states. Otherwise, if a frame (e.g., Z-then-X or Z-then-bisector) or its frame-defining atoms at end states differ, then the torque contributions need to be collected separately (e.g., the multipolar contributions from HIS, HID, and HIE end-states would need to be rotated into the global frame separately and their respective torques accumulated separately). Therefore, all permanent atomic multipoles impacted by titration were made consistent with their counterpart(s). Fortunately, this affected only a few titration sites for the carboxylic acids and cysteine (see the updated AMOEBA protein force field parameters in the Supporting Information). Analogous to permanent atomic multipole frame definitions that require consistency, polarization groups for each atomic site must also concur. The permanent multipoles of atoms that belong to the same polarization group do not contribute to the field that induces the dipoles of other atoms within their group. If there is a difference in how the polarization groups are defined between titration end states, then the direct field cannot be defined in a way that is consistent with the use of a single self-consistent field calculation. Fortunately, the only polarization group that required modification was for the deprotonated cysteine.

Finally, an additional small approximation to simplify the algorithm was to consider the bonded terms invariant to titration variables, which was also made in previous work.^28,29,34^ This was achieved by fixing the bonded terms to those of the protonated state. In the case of carboxylic acids, force field terms were defined as if both oxygen atoms were bonded to a “dummy” hydrogen. Over the course of a simulation, the carboxylic acid could have only one hydrogen atom present with full-strength nonbonded interactions; however, both oxygen atoms always maintain a hydrogen via standard bonded terms.

### pH Replica Exchange

Due to the computational expense of CpHMD simulations in the AMOEBA force field, a pH-based replica exchange (pH-REX) protocol^44^ was implemented to accelerate convergence through enhanced sampling. The protocol involves running multiple simulations at different pH values simultaneously and periodically exchanging pH’s between simulations according to a probability given by the Metropolis criterion
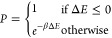
49where β is given by 1/(*k*_B_*T*) and Δ*E* is defined as
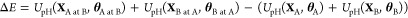
50where A and B represent the two ensembles considered in the exchange.

The force exerted on a proton in response to a new pH that occurs after an exchange often encourages the titration-tautomer coordinates to overcome energetic barriers. This enhances the sampling by allowing for more frequent barrier crossing than would be possible in a simulation with a single fixed pH. This protocol also lends itself well to the generation of titration curves, where each pH window in the replica exchange contributes to the titration curve. For the predictions of the model and protein p*K*_a_ values, an exchange was attempted every 500 molecular dynamics steps.

## Simulation Protocol

### Crystal Peptide System Preparation

All systems were set up and simulated in the open-source Force Field X (FFX) software package. Ten previously studied crystal peptides were obtained from the Cambridge Structural Database^45^ in CIF format. Their database IDs are as follows: RAVZAQ, RAVZEU, RAVZIY, RAVZOE, RAVZUK, RAWBAT,^46^ JUKMOR,^47^ TEKNAY,^48^ CURLOQ,^49^ and CUFFUG.^50^ Each structure had protons added to each titratable site, and a *P*1 system was prepared in addition to the asymmetric unit (excluding CURLOQ due to reasons mentioned below). Conversion from CIF format to Tinker XYZ format^51^ with atom types was facilitated by the FFX command *ImportCIF*.

### Crystal Peptide CpHMD Production Simulations

The asymmetric units and unit cells were set up according to the deposited crystal records through FFX’s capability to consistently handle symmetry for all space groups with PME.^43^ The asymmetric system experienced the same crystal packing as the *P*1 system except for having fewer independently titrating sites. Langevin dynamics simulations, including fluctuating protonation states, were completed at a neutral pH of 7.0 for 10 ns in the NVT ensemble. The temperature was set to 193 K to match the conditions of the previously studied crystals.^46^ Each of the titratable sites was started at an intermediate state (*λ*_*k*_ = 0.5, *ζ*_*k*_ = 0.5) to facilitate the titration state quickly sampling an equilibrium value. PME was used for electrostatics with a cutoff of 7 Å, an Ewald alpha of 0.545, and a neutrality constraint.^52^ For van der Waals interactions, a switching function was used to smoothly switch off interactions over a window of 10 to 12 Å.

### Peptide Model System Preparation

Capped pentapeptides were used as the model compounds for the determination of the model bias for each residue (eq 4) and took the form of Ace-AAXAA-Nme (X = ASP, GLU, HIS, LYS, CYS). The model peptide compounds were solvated in a 40 Å box of explicit water, where the padding of water was at least 10 Å. Finally, the solvated systems were minimized using the L-BFGS algorithm.^53^ All atoms were represented using the AMOEBA-BIO-2018 force field^54^ with the modified parameters discussed above and given in the Supporting Information.

### Model Parameterization

Model parametrization began by collecting the potential of mean force (PMF) curves for the titration of each model compound. This was accomplished by running 50 ns of Langevin dynamics for each of 11 evenly spaced titration windows spanning λ = 0 (deprotonated) to λ = 1 (protonated). The conditions for the dynamics were NVT at 298 K. In each of these windows, the λ value is fixed so that only conformational sampling is collected (i.e., the titration/tautomer state does not fluctuate). The Bennet acceptance ratio method^55^ was then used to estimate the free-energy difference between neighboring windows, and the resulting PMF was fit to the polynomial model bias equation (eq 4). As mentioned above, HIS required three such PMFs to be generated. Two titration PMFs were generated at fixed tautomer values of 0 and 1 to collect the PMF of protonating HID and HIE, respectively. A third PMF was generated to shift the proton from δ-N to ϵ-N by running dynamics over tautomer windows (rather than titration). Once these PMFs were in place as model bias terms, they were validated against three replicate pH-replica exchange molecular dynamics simulations. Titration curves for the model compounds were generated, and the predicted p*K*_a_ for the models was assessed. The titration curve was generated from a pH range centered on the p*K*_a_ of the model and separated by 0.5 pH unit by using 10 ns simulations. The Langevin dynamics for each replica were carried out under NVT conditions using a 1 fs time step at 298 K. PME was used for electrostatics with a cutoff of 7 Å. Lennard-Jones interactions were switched off over the range of 10–12 Å. Although the forces due to the titration or tautomer variables cannot yet be calculated on a GPU, a hybrid scheme has been adopted to achieve some acceleration on the protein simulations. This scheme involves cycling MD steps between the CPU and GPU. On the CPU, the full sets of forces can be calculated, so the titration states and conformational states can be updated simultaneously. Once a desired number of steps has been reached, the CPU can send the interpolated multipoles and polarizabilities to OpenMM^56^ to perform conformational dynamics on these “frozen” titration states. No changes to OpenMM were required because interpolated multipole and polarizability values were handled analogously to the standard AMOEBA force field parameters for amino acid end states. This permits continuous titration states while avoiding the need for Metropolis Monte Carlo moves between discrete end states. After OpenMM completed a specified number of steps, the CPU continues from the new coordinates to begin the next cycle (see the Supporting Information for a detailed description). Although full acceleration through GPUs is a future goal, this scheme has allowed more efficient sampling convergence than CPU moves alone without requiring the OpenMM AMOEBA plugin code to be modified.

### p*K*_a_ Calculation

Over the course of the simulations, the λ coordinates are saved. Any λ value that is ≤0.10 or ≥0.90 is used to calculate the respective (de)protonated fractions. For λ ≤ 0.10, the state is considered to be deprotonated, and for λ ≥ 0.90, it is considered to be protonated. Values that do not meet these criteria are discarded. The deprotonated fraction was calculated and fit to the Hill equation^28,29^
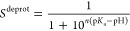
51where p*K*_a_ and *n* are fit parameters and *n* represents the Hill coefficient. The deprotonated fraction can be defined by simulation counts as *S*^deprot^ = *N*_deprot_/(*N*_deprot_ + *N*_prot_)), where *N* is the number of counts for the respective deprotonated or protonated state.

### Experimental Protocol

A crystal structure was generated for this work of an l-histidyl-l-alanine (HIS-ALA) dipeptide in an attempt to further analyze a structure from a previous study.^48^ The compound was purchased from Ambeed, Inc. and was received as a salt with trifluoroacetic acid (TFA) instead of the desired pure form. It was crystallized by the vapor diffusion of ethanol into an aqueous solution of dipeptide using the hanging drop method. The experiment was set up in a 24-well plate wherein 1000 μL of ethanol was added to each well and 2 μL of concentrated aqueous solution of dipeptide was placed on the coverslip. The coverslip was placed in an inverted position on the well, and the walls of the well were sealed with grease. The plate was kept in a vibration-free zone until single crystals suitable for XRD were visible.

### X-ray Crystallography

Data were collected on a Bruker D8 VENTURE DUO diffractometer equipped with an IμS 3.0 microfocus source operated at 55 W (50 kV, 1.1 mA) to generate Cu Kα radiation (λ = 1.54178 Å) and a PHOTON III detector. The crystal was maintained at 100 K throughout the duration of the experiment. Data collection, initial indexing, and final cell parameter calculations were carried out using APEX4.^57^ These values are reported in the Results and Discussion. A numerical absorption correction was applied based on a Gaussian integration over a multifaceted crystal and followed by a semiempirical correction for adsorption applied using SADABS.^57^ The program SHELXT^58^ was used for the initial structure solution, and SHELXL^59^ was used for the refinement of the structure. Both of these programs were utilized within the OLEX2 software.^60^ Hydrogen atoms bound to carbon atoms were located in the difference Fourier map and geometrically constrained by using the appropriate AFIX commands. The hydrogen atoms bound to the nitrogen atoms were allowed to freely refine their atomic positions.

## Results and Discussion

### Pentapeptide Titration Curves

**Figure 1:**
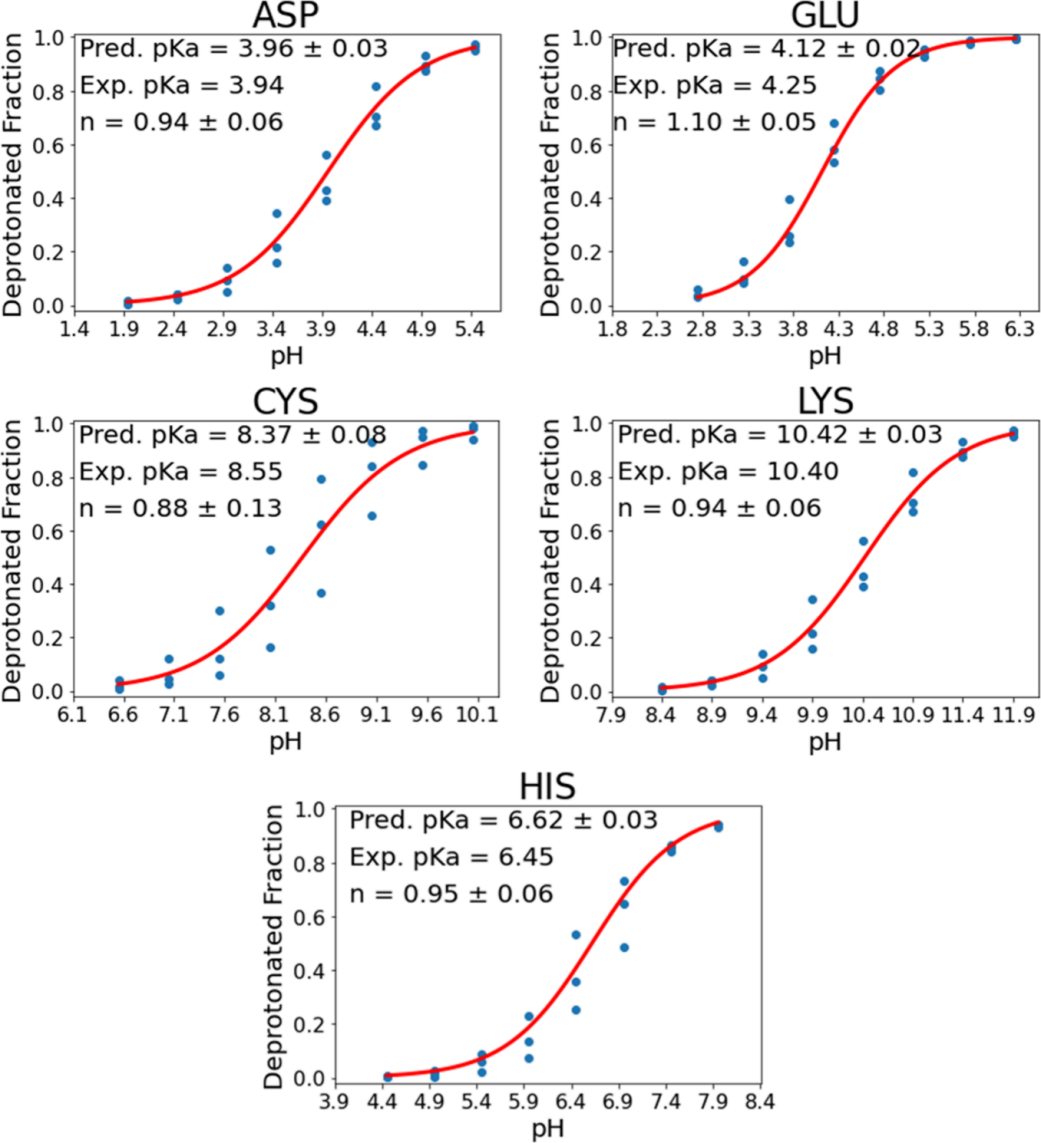
Titration curves are generated for each pentapeptide. Listed for each are the predicted p*K*_a_ value, the experimental value, and the Hill coefficient.

Titration curves for the model pentapeptides were generated and used to assess the model PMF determined from BAR simulations as described in the methods. If the PMF is appropriately sampled and subtracted during constant-pH dynamics, then the predicted p*K*_a_ should match the reference value used in the model. This is validated by fitting the unprotonated fractions to eq 51 to determine the p*K*_a_. The unprotonated fractions are converged using pH replica exchange simulations over a range of pH values described above. The predicted values can be seen in Figure 1 along with experimental values. The Hill coefficient is also measured from eq 51 to evaluate the deviation from the standard Henderson–Hasselbalch curvature. In the case of a single titrating site, deviations far from unity reflect the response of the model rather than cooperative effects. Many-body polarization of the AMOEBA energy function influences the shape of the titration curve in a manner that differs from prior CpHMD models based on fixed partial charge fields. For example, unlike the AMOEBA model for oxygen or nitrogen, the polarizability of the CYS sulfur atom changes from 4.0 to 2.8 Å^3^ as it is protonated.

### HIS-ALA TFA Crystal Results

**Figure 2:**
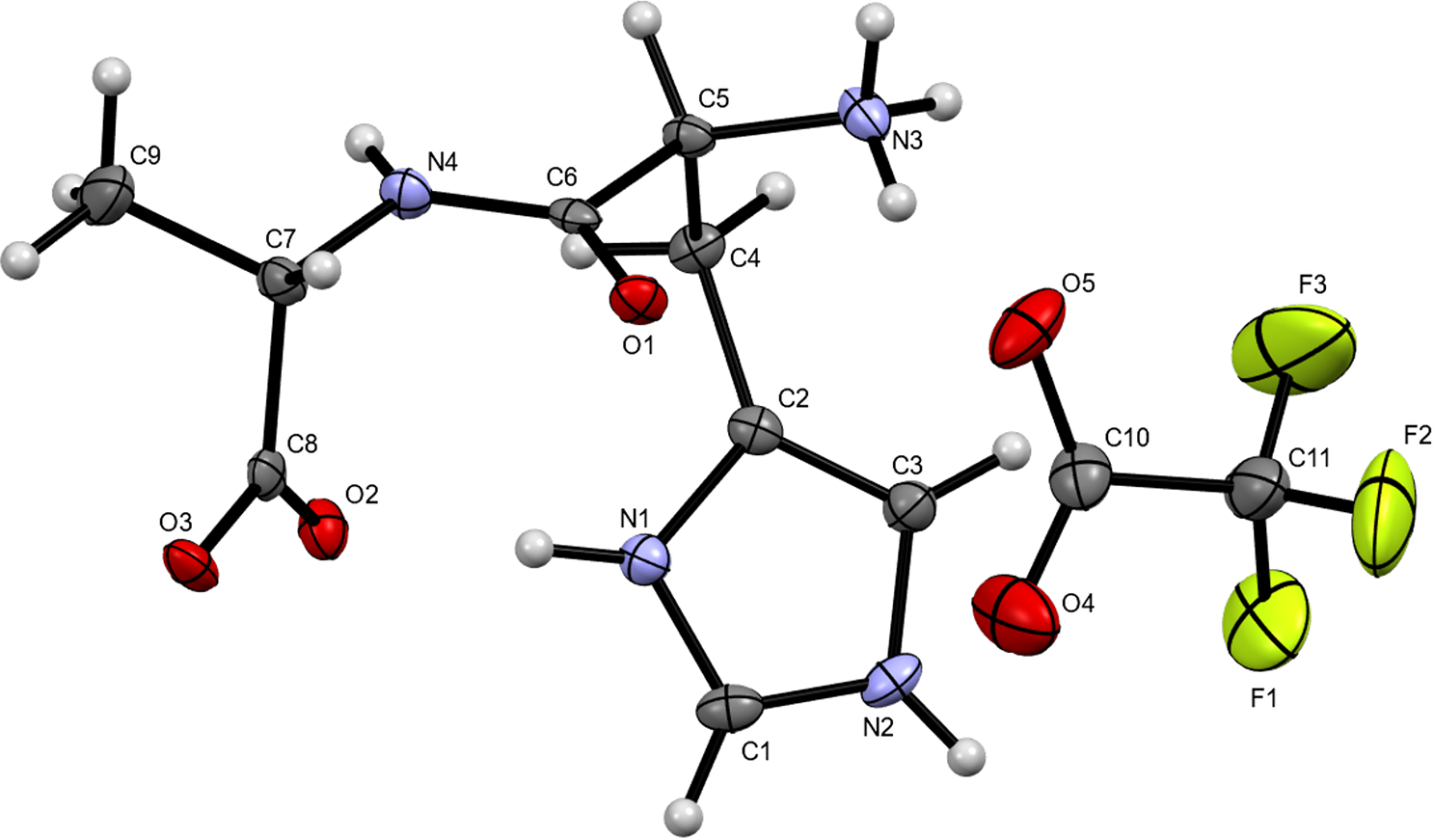
Thermal ellipsoids are represented at 50% probability. Carbon, hydrogen, nitrogen, oxygen, and fluorine atoms are represented by gray, white, light-blue, red, and light-green ellipsoids, respectively.

The HIS-ALA dipeptide crystallized as a zwitterion, and the refinement data can be seen in Table 2. The dipeptide was cocrystallized with one molecule of TFA. The TFA was observed to be deprotonated, and the histidine ring was observed to be protonated as seen in Figure 2.

**Table 2:** Crystal Refinement Parameters for HIS-ALA TFA

Identification code	CCDC no. 2292810		ρ_calc_g/cm^3^	1.554
Empirical formula	C_11_H_15_F_3_N_4_O_5_		μ/mm^–1^	1.294
Formula weight	340.27		F(000)	704
Temperature/K	100		Crystal size/mm^3^	0.138 × 0.075 × 0.047
Crystal system	orthorhombic		Radiation	Cu Kα (λ = 1.54178)
Space group	*P2*_*1*_*2*_*1*_*2*_*1*_		2Θ range for data collection/deg	8.024 to 144.346
*a*/Å	5.5240(2)		Index ranges	–6 ≤ *h* ≤ 6, –16 ≤ *k* ≤ 16, –24 ≤ *l* ≤ 22
*b*/Å	13.2352(4)		Reflections collected	17095
*c*/Å	19.8946(5)		Independent reflections	2875 [*R*_int_ = 0.0612, *R*_σ_ = 0.0391]
α/deg	90		Data/restraints/parameters	2875/0/227
β/deg	90		Goodness of fit on F^2^	1.064
γ/deg	90		Final R indexes [I ≥ 2σ(I)]	R_1_ = 0.0391, wR_2_ = 0.0996
Volume/Å^3^	1454.52(8)		Final R indexes [all data]	R_1_ = 0.0428, wR_2_ = 0.1021
Z	4		Largest diff. peak/hole/e Å^–3^	0.38/–0.29
			Flack parameter	–0.13(11)

### Crystal Peptide Protonation State Predictions

**Figure 3:**
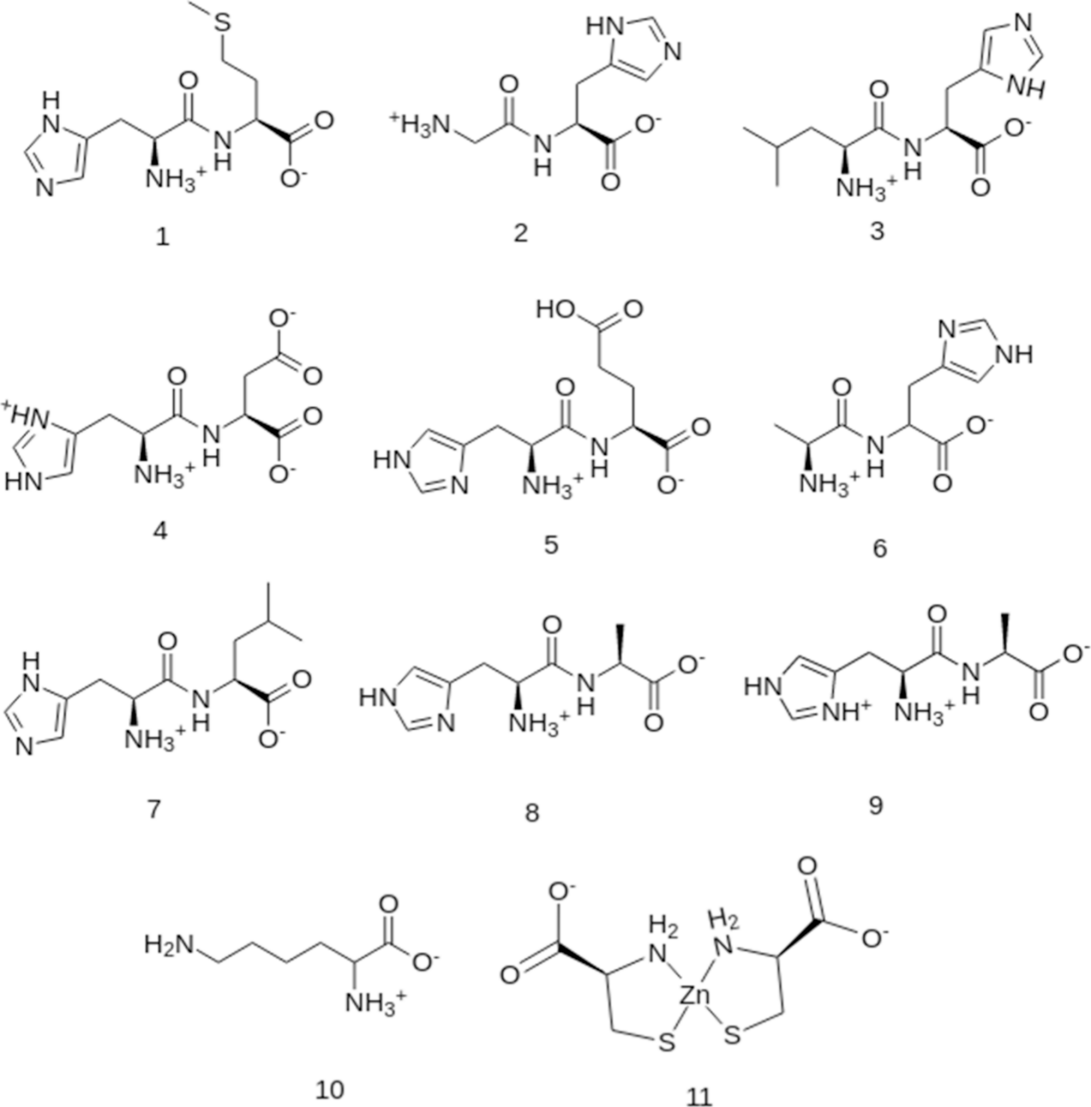
Structures of the simulated crystal peptides: (1) RAVZAQ (HID-MET), (2) RAVZEU (GLY-HID), (3) RAVZIY (LEU-HID), (4) RAVZOE (HIS-ASP), (5) RAVZUK (HIE-GLH), (6) RAWBAT (ALA-HIE), (7) JUKMOR (HID-LEU), (8) TEKNAY (HIE-ALA), (9) this work (CCDC no. 2292810) (HIS-ALA), (10) CUFFUG (LYS), and (11) CURLOQ (CYD-Zn-CYD).

Eight of the 11 crystal peptide structures were investigated previously to shed light on the different NMR chemical shifts for the two histidine tautomer states.^46^ The tautomeric composition of these eight structures consists of four crystals with the HID state (hydrogen atom present at δ-*N*), three with the HIE state (hydrogen atom present at ε-*N*,), and one in the charged HIS state. The varied nature of these histidine-containing peptides is ideal for validating this method’s ability to distinguish the histidine tautomer states from one another. The data set also includes the carboxylic acid residues, making it a useful target for evaluating the accuracy in determining coupled protonation states (i.e., where the crystal may be either a salt or a cocrystal). The remaining two crystal peptides cover lysine and cysteine residues that are not present in the prior study that focused on histidine. The chemical structures for the 11 peptides can be seen in Figure 3. The cysteine crystal includes zinc, which is coordinated by two sulfur groups and two amino groups. This is particularly interesting as a model of zinc coordination in proteins, where zinc is often coordinated by cysteine.

**Figure 4:**
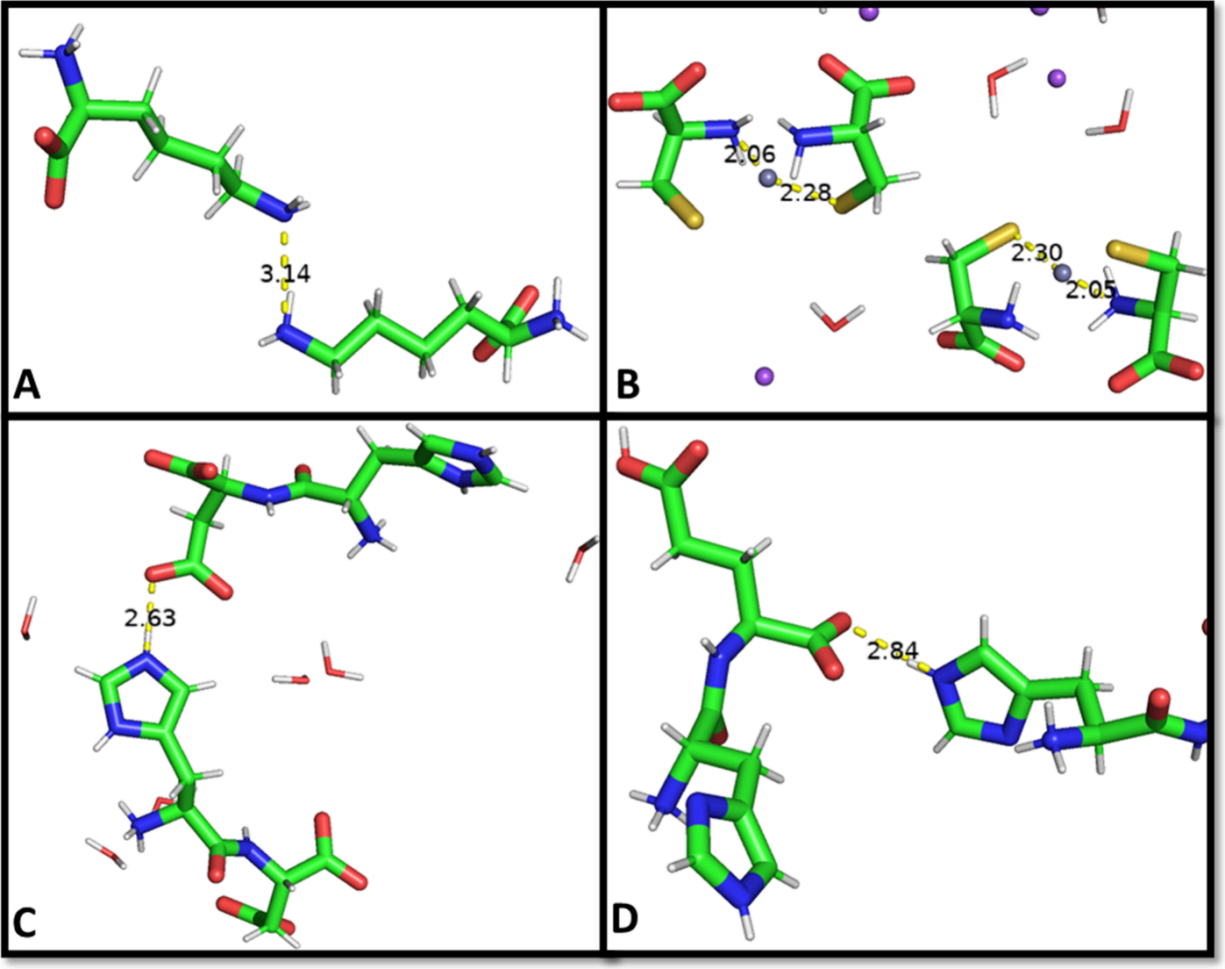
Representative interatomic distances for 4 of the 11 dipeptides encompassing each functional group titrated in the AMOEBA CpHMD simulations. (A) Interatomic distance between the nitrogens of lysine peptides in CUFFUG is shown. (B) CURLOQ system is shown with the interatomic distance between the sulfur of the cysteine side chain and the zinc ion. (C, D) Distance between the titrating ε-nitrogen of histidine and either the aspartate side chain of RAVZOE in the first case or the oxygen of the C-terminus of RAVZUK in the second case.

As can be seen in Table 3, the AMOEBA constant-pH algorithm performs well in selecting the correct titration and tautomer states while maintaining close agreement with the experimental structure, as seen in Table 4. Close attention was paid to the area around the titrating site, and no significant disturbances were found between polar contacts. Examples of this can be seen in Figure 4.

**Table 3:** The 11 Crystal Peptides Examined in This Study^a^

CCDC ID. (Exp. Titration State)	Space Group	Coformers	Asymmetric Unit Independent Predictions	Unit Cell Independent Predictions	Observed Fractions
**RAVZAQ** (HID-MET)	*P*2_1_	None	1	2	HID: >0.99
**RAVZEU** (GLY-HID)	*P*2_1_	Dihydrate	1	2	HID: >0.99
**RAVZIY** (LEU-HID)	*P*2_1_2_1_2	Monohydrate	1	4	HID: >0.99
**RAVZOE** (HIS-ASP)	*P*2_1_	Trihydrate	2	4	HIS: >0.99
					ASP: 0.98
**RAVZUK** (HIE-GLH)	*P*2_1_	None	2	4	HIE: >0.99
					GLH: 0.99
**RAWBAT** (ALA-HIE)	*P*2_1_^b^	Ethanol solvate hemihydrate	2	4	HIE: >0.99
**JUKMOR** (HID-LEU)	*P*2_1_	None	1	2	HID: >0.99
**TEKNAY** (HIE-ALA)	*P*2_1_	Dihydrate	1	2	HID: 0.50
					HIE: 0.49
***This Work*** (HIS-ALA)	*P*2_1_2_1_2_1_	Trifluoroacetic acid	1	4	HIS: >0.99
**CUFFUG** (LYD)	*P*2_1_	None	2	4	LYD: 0.83
					Inter: 0.17
**CURLOQ** (CYD)	*C*2	Hexahydrate, tetrasodium	N.A.	8	CYD: >0.99
**Total Predictions**			14	40	

a The first column lists each crystal’s accession ID from the CCDC, followed by its space group in the second column. The third column lists coformer(s) present in the crystal along with the peptide of interest. In the fourth and fifth columns, the number of independent observations in the asymmetric unit and unit cell, respectively, are given. In the final column is the three-letter shorthand for the experimental titration state(s) and the observed titration state fractions from simulation. In the reported fractions, “Inter” represents the fraction of samples observed in an intermediate alchemical lambda state that is not considered to be consistent with a physical end-state. This value is listed only for the case of CUFFUG (LYD), where it was greater than for 1% of the samples.

b The reported space group for CCDC ID RAWBAT is *P*2_1_2_1_2; however, it was expanded to *P*2_1_ for our simulations of the asymmetric unit. The asymmetric unit could not be appropriately simulated for CURLOQ due to the presence of atoms with fractional occupancy.

**Table 4:** Coordinate RMSDs and Distance Comparisons for the 11 Studied Peptides^a^

CCDC ID (Exp. Titration State)	Titration Site to Nearest Polar Contact	Experimental Distance (Å)	Mean Simulated Distance (Å)	RMSD (Å)
**RAVZAQ** (HID-MET)	Histidine-Nδ−O-C_terminus_	2.72	2.96 ± 0.10	0.18 ± 0.03
**RAVZEU** (GLY-HID)	Histidine-Nδ−O-C_terminus_	2.88	2.98 ± 0.09	0.16 ± 0.04
**RAVZIY** (LEU-HID)	Histidine-Nδ−O-C_terminus_	2.73	2.90 ± 0.08	0.26 ± 0.05
**RAVZOE** (HIS-ASP)	Histidine-Nε–O-Aspartate	2.63	2.85 ± 0.11	0.56 ± 0.10
**RAVZUK** (HIE-GLH)	Histidine-Nε–O-C_terminus_	2.84	2.88 ± 0.07	0.19 ± 0.04
**RAWBAT** (ALA-HIE)	Histidine-Nε–O-Water	2.87	2.97 ± 0.13	0.14 ± 0.03
**JUKMOR** (HID-LEU)	Histidine-Nδ−O-C_terminus_	2.71	2.95 ± 0.09	0.26 ± 0.08
**TEKNAY** (HIE-ALA)	Histidine-Nδ−O-Water	2.80	2.86 ± 0.07	0.24 ± 0.08
***This Work*** (HIS-ALA)	Histidine-Nε–O-C_terminus_	2.66	2.76 ± 0.09	0.78 ± 0.17
**CUFFUG** (LYD)	Lysine-Nζ–Nζ-Lysine	3.14	3.64 ± 0.48	0.60 ± 0.09
**CURLOQ** (CYD)	Cysteine-S–Zn^2+^ ion	2.28	2.16 ± 0.04	0.38 ± 0.07

a The RMSDs were calculated using the progressive alignment of crystals (PAC) method,^61^ with a single peptide molecule used for the comparison. Coformers were ignored in the RMSD comparison. Interatomic distance comparisons were taken between the titrating heavy atom and its nearest polar contact, which may also be a titrating heavy atom. The standard deviations are based on the fluctuations observed during CpHMD simulations.

It is encouraging to see HID predictions that match experiment in each of the four cases, considering that in water, the HIE tautomer state should be favored over HID roughly 70% of the time when histidine is deprotonated. The CpHMD AMOEBA algorithm matched the experiment in selecting the histidyl aspartate crystal as a salt and the histidyl glutamate as a cocrystal with the correct tautomer state. The prediction of deprotonated lysine and cysteine titration states confirms that the model responds to net charge. Convergence was assessed from the separate asymmetric unit and *P*1 simulations. For all but TEKNAY, each system displayed a rapid convergence to the experimentally observed state. The asymmetric unit and *P*1 systems were nearly identical, and the results across the independently titrating sites in the *P*1 systems were also nearly identical. Each system converged within a few hundred picoseconds as demonstrated in the time series for the HIS-ALA TFA given in Figure S1 of the Supporting Information. This can also be observed from the populations given in Table 3.

**Figure 5:**
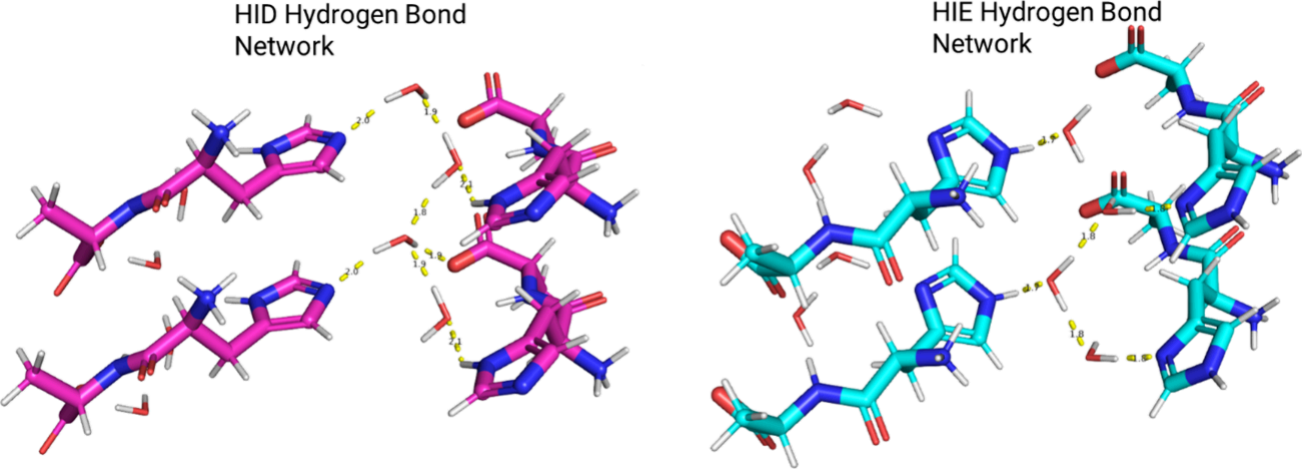
Alternative hydrogen bonding networks for histidyl alanine dihydrate tautomers (CCDC ID: TEKNAY) observed during the CpHMD simulations.

The only compound for which the prediction did not strictly match the experiment was for the histidyl alanine dihydrate, TEKNAY. For this dipeptide, the CpHMD simulations sampled both the HID and HIE tautomer states in significant populations. Over the course of the simulation, the histidine frequently sampled both deprotonated tautomer states, HID and HIE, with a slight preference for HID. An analysis of the trajectory showed that each tautomer state established a hydrogen bonding network with its surroundings that required minor conformational adjustments of the water molecules. As can be seen in Figure 5, the relative oxygen positions of the water molecules moved only a small amount to accommodate the reorientation of the hydrogen bond network. The distances between hydrogen bond donors and acceptors are also comparable. These observations suggest that both states are physically accessible and similar in energy. This is further supported by a free-energy assessment. Using the Rao–Blackwell estimator for free-energy differences as laid out by Ding et al.,^62^ the free-energy difference between the two states from a 40 ns trajectory was calculated to be only 0.35 ± 0.03 kcal/mol.

## Conclusions

We presented the first implementation, parametrization, and validation of a continuous constant-pH molecular dynamics algorithm using the multipolar polarizable AMOEBA force field under PME. Parameters were derived for the pentapeptides (Ace-AA-X-AA-Nme, where X is the residue of interest) for ASP, GLU, HIS, LYS, and CYS. These parameters were validated by constructing titration curves for each pentapeptide using the CpHMD algorithm accelerated with pH replica exchange. Further validation was performed on a set of 11 crystal peptides, 10 previously studied and 1 generated here. These crystal peptides provide a novel test suite for CpHMD methods using PME electrostatics because their atomic coordinates and titration states are generally well-defined. Each crystal was simulated as an asymmetric unit using FFX’s unique capability to handle symmetry operators as well as in the expanded unit cells (*P*1). The results were exactly replicated between the asymmetric unit and unit cell systems. The experimental titration states observed for each crystal peptide were reproduced for all except one crystal. Our simulations, supported by a free-energy difference estimate, indicated that disorder was present between the two tautomeric states of histidine for the histidyl alanine dihydrate crystal (CCDC ID: TEKNAY). Further investigation of this crystal system is ongoing, including recrystallization and refinement. The success of this algorithm in predicting titration states of peptides in a crystalline environment using the traditional parametrization of a peptide in water suggests that this model will be suitable for p*K*_a_ prediction in proteins under PME. Future work will incorporate CpHMD into AMOEBA protein simulations based on the generalized Kirkwood implicit solvent.^63,64^

## Data Availability

Constant-pH molecular dynamics trajectories generated during this work can be regenerated using Force Field X (https://github.com/SchniedersLab/forcefieldx, https://ffx.biochem.uiowa.edu/).

## Supporting Information Available

Functional form for each extended Hamiltonian equation and their parameters for the titrating functional groups; exact modifications to the AMOEBA protein force field parameters; and various figures providing additional context to the titration curves and convergence of the crystal simulations (PDF)

## References

[ref1] Elcock A. H.; McCammon J. A. Electrostatic contributions to the stability of halophilic proteins. Journal of molecular biology 1998, 280 (4), 731–748. 10.1006/jmbi.1998.1904. 9677300

[ref2] Creighton T. E. Proteins: Structures and Molecular Properties, 2nd ed.; W. H. Freeman: New York, 1993.

[ref3] Dobson C. M. Protein folding and misfolding. Nature 2003, 426 (6968), 884–890. 10.1038/nature02261. 14685248

[ref4] Haas J.; Vohringer-Martinez E.; Bogehold A.; Matthes D.; Hensen U.; Pelah A.; Abel B.; Grubmuller H. Primary steps of pH-dependent insulin aggregation kinetics are governed by conformational flexibility. Chembiochem 2009, 10 (11), 1816–1822. 10.1002/cbic.200900266. 19533727

[ref5] Entsch B.; Cole L. J.; Ballou D. P. Protein dynamics and electrostatics in the function of p-hydroxybenzoate hydroxylase. Archives of biochemistry and biophysics 2005, 433 (1), 297–311. 10.1016/j.abb.2004.09.029. 15581585

[ref6] Chenprakhon P.; Panijpan B.; Chaiyen P. An experiment illustrating the change in ligand p K a upon protein binding. J. Chem. Educ. 2012, 89 (6), 791–795. 10.1021/ed2006482.

[ref7] Highbarger L. A.; Gerlt J. A.; Kenyon G. L. Mechanism of the reaction catalyzed by acetoacetate decarboxylase. Importance of lysine 116 in determining the p K a of active-site lysine 115. Biochemistry 1996, 35 (1), 41–46. 10.1021/bi9518306. 8555196

[ref8] Stouffer A. L.; Acharya R.; Salom D.; Levine A. S.; Di Costanzo L.; Soto C. S.; Tereshko V.; Nanda V.; Stayrook S.; DeGrado W. F. Structural basis for the function and inhibition of an influenza virus proton channel. Nature 2008, 451 (7178), 596–599. 10.1038/nature06528. 18235504 PMC3889492

[ref9] Tournaire-Roux C.; Sutka M.; Javot H.; Gout E.; Gerbeau P.; Luu D. T.; Bligny R.; Maurel C. Cytosolic pH regulates root water transport during anoxic stress through gating of aquaporins. Nature 2003, 425 (6956), 393–397. 10.1038/nature01853. 14508488

[ref10] Wiley D. C.; Skehel J. J. The structure and function of the hemagglutinin membrane glycoprotein of influenza virus. Annu. Rev. Biochem. 1987, 56, 365–394. 10.1146/annurev.bi.56.070187.002053. 3304138

[ref11] Manallack D. T.; Prankerd R. J.; Yuriev E.; Oprea T. I.; Chalmers D. K. The significance of acid/base properties in drug discovery. Chem. Soc. Rev. 2013, 42 (2), 485–496. 10.1039/C2CS35348B. 23099561 PMC3641858

[ref12] Pracht P.; Grimme S. Efficient quantum-chemical calculations of acid dissociation constants from free-energy relationships. J. Phys. Chem. A 2021, 125 (25), 5681–5692. 10.1021/acs.jpca.1c03463. 34142841

[ref13] Haslak Z. P.; Zareb S.; Dogan I.; Aviyente V.; Monard G. Using Atomic Charges to Describe the p K a of Carboxylic Acids. J. Chem. Inf. Model. 2021, 61 (6), 2733–2743. 10.1021/acs.jcim.1c00059. 34137248

[ref14] Olsson M. H.; Søndergaard C. R.; Rostkowski M.; Jensen J. H. PROPKA3: consistent treatment of internal and surface residues in empirical p K a predictions. J. Chem. Theory Comput. 2011, 7 (2), 525–537. 10.1021/ct100578z. 26596171

[ref15] Søndergaard C. R.; Olsson M. H.; Rostkowski M.; Jensen J. H. Improved treatment of ligands and coupling effects in empirical calculation and rationalization of p K a values. J. Chem. Theory Comput. 2011, 7 (7), 2284–2295. 10.1021/ct200133y. 26606496

[ref16] Reis P. B.; Vila-Vicosa D.; Rocchia W.; Machuqueiro M. PypKa: A Flexible Python Module for Poisson-Boltzmann-Based p K a Calculations. J. Chem. Inf. Model. 2020, 60 (10), 4442–4448. 10.1021/acs.jcim.0c00718. 32857502

[ref17] Reis P. B.; Bertolini M.; Montanari F.; Rocchia W.; Machuqueiro M.; Clevert D.-A. A Fast and Interpretable Deep Learning Approach for Accurate Electrostatics-Driven p K a Predictions in Proteins. J. Chem. Theory Comput. 2022, 18 (8), 5068–5078. 10.1021/acs.jctc.2c00308. 35837736 PMC9369009

[ref18] de Oliveira V. M.; Liu R.; Shen J. Constant ph molecular dynamics simulations: current status and recent applications. Curr. Opin. Struct. Biol. 2022, 77, 102498 10.1016/j.sbi.2022.102498. 36410222 PMC9933785

[ref19] Baptista A. M.; Martel P. J.; Petersen S. B. Simulation of protein conformational freedom as a function of pH: constant-pH molecular dynamics using implicit titration. Proteins: Struct., Funct., Bioinf. 1997, 27 (4), 523–544. 10.1002/(SICI)1097-0134(199704)27:4<523::AID-PROT6>3.0.CO;2-B. 9141133

[ref20] Baptista A. M.; Teixeira V. H.; Soares C. M. Constant-p H molecular dynamics using stochastic titration. J. Chem. Phys. 2002, 117 (9), 4184–4200. 10.1063/1.1497164.

[ref21] Metropolis N.; Rosenbluth A. W.; Rosenbluth M. N.; Teller A. H.; Teller E. Equation of state calculations by fast computing machines. J. Chem. Phys. 1953, 21 (6), 1087–1092. 10.1063/1.1699114. 41342507

[ref22] Mongan J.; Case D. A.; McCammon J. A. Constant pH molecular dynamics in generalized Born implicit solvent. Journal of computational chemistry 2004, 25 (16), 2038–2048. 10.1002/jcc.20139. 15481090

[ref23] Bürgi R.; Kollman P. A.; Van Gunsteren W. F. Simulating proteins at constant pH: An approach combining molecular dynamics and Monte Carlo simulation. Proteins: Struct., Funct., Bioinf. 2002, 47 (4), 469–480. 10.1002/prot.10046. 12001225

[ref24] Meng Y.; Roitberg A. E. Constant pH replica exchange molecular dynamics in biomolecules using a discrete protonation model. J. Chem. Theory Comput. 2010, 6 (4), 1401–1412. 10.1021/ct900676b. 20514364 PMC2877402

[ref25] Swails J. M.; York D. M.; Roitberg A. E. Constant pH replica exchange molecular dynamics in explicit solvent using discrete protonation states: implementation, testing, and validation. J. Chem. Theory Comput. 2014, 10 (3), 1341–1352. 10.1021/ct401042b. 24803862 PMC3985686

[ref26] Chen Y.; Roux B. Constant-pH hybrid nonequilibrium molecular dynamics-Monte Carlo simulation method. J. Chem. Theory Comput. 2015, 11 (8), 3919–3931. 10.1021/acs.jctc.5b00261. 26300709 PMC4535364

[ref27] Radak B. K.; Chipot C.; Suh D.; Jo S.; Jiang W.; Phillips J. C.; Schulten K.; Roux B. Constant-pH molecular dynamics simulations for large biomolecular systems. J. Chem. Theory Comput. 2017, 13 (12), 5933–5944. 10.1021/acs.jctc.7b00875. 29111720 PMC5726918

[ref28] Lee M. S.; Salsbury F. R. Jr; Brooks III C. L. Constant-pH molecular dynamics using continuous titration coordinates. Proteins: Struct., Funct., Bioinf. 2004, 56 (4), 738–752. 10.1002/prot.20128. 15281127

[ref29] Khandogin J.; Brooks C. L. Constant pH molecular dynamics with proton tautomerism. Biophysical journal 2005, 89 (1), 141–157. 10.1529/biophysj.105.061341. 15863480 PMC1366513

[ref30] Donnini S.; Tegeler F.; Groenhof G.; Grubmüller H. Constant pH Molecular Dynamics in Explicit Solvent with λ-Dynamics. J. Chem. Theory Comput. 2011, 7 (6), 1962–1978. 10.1021/ct200061r. 21687785 PMC3114466

[ref31] Goh G. B.; Knight J. L.; Brooks C. L. Constant pH Molecular Dynamics Simulations of Nucleic Acids in Explicit Solvent. J. Chem. Theory Comput. 2012, 8 (1), 36–46. 10.1021/ct2006314. 22337595 PMC3277849

[ref32] Huang Y.; Chen W.; Wallace J. A.; Shen J. All-atom continuous constant pH molecular dynamics with particle mesh Ewald and titratable water. J. Chem. Theory Comput. 2016, 12 (11), 5411–5421. 10.1021/acs.jctc.6b00552. 27709966 PMC5713900

[ref33] Huang Y.; Harris R. C.; Shen J. Generalized Born based continuous constant pH molecular dynamics in Amber: Implementation, benchmarking and analysis. J. Chem. Inf. Model. 2018, 58 (7), 1372–1383. 10.1021/acs.jcim.8b00227. 29949356 PMC6516748

[ref34] Harris J. A.; Liu R.; Martins de Oliveira V.; Vázquez-Montelongo E. A.; Henderson J. A.; Shen J. GPU-accelerated all-atom particle-mesh Ewald continuous constant pH molecular dynamics in Amber. J. Chem. Theory Comput. 2022, 18 (12), 7510–7527. 10.1021/acs.jctc.2c00586. 36377980 PMC10130738

[ref35] Wallace J. A.; Shen J. K. Charge-leveling and proper treatment of long-range electrostatics in all-atom molecular dynamics at constant pH. J. Chem. Phys. 2012, 137 (18), 184105 10.1063/1.4766352. 23163362 PMC3511335

[ref36] Chen W.; Wallace; Jason A.; Yue Z.; Shen; Jana K. Introducing Titratable Water to All-Atom Molecular Dynamics at Constant pH. Biophys. J. 2013, 105 (4), L15–L17. 10.1016/j.bpj.2013.06.036. 23972860 PMC3752133

[ref37] Schnieders M. J.; Baltrusaitis J.; Shi Y.; Chattree G.; Zheng L.; Yang W.; Ren P. The structure, thermodynamics, and solubility of organic crystals from simulation with a polarizable force field. J. Chem. Theory Comput. 2012, 8 (5), 1721–1736. 10.1021/ct300035u. 22582032 PMC3348590

[ref38] Ren P.; Ponder J. W. Polarizable atomic multipole water model for molecular mechanics simulation. J. Phys. Chem. B 2003, 107 (24), 5933–5947. 10.1021/jp027815+.

[ref39] Ren P.; Wu C.; Ponder J. W. Polarizable atomic multipole-based molecular mechanics for organic molecules. J. Chem. Theory Comput. 2011, 7 (10), 3143–3161. 10.1021/ct200304d. 22022236 PMC3196664

[ref40] Shi Y.; Xia Z.; Zhang J.; Best R.; Wu C.; Ponder J. W.; Ren P. Polarizable atomic multipole-based AMOEBA force field for proteins. J. Chem. Theory Comput. 2013, 9 (9), 4046–4063. 10.1021/ct4003702. 24163642 PMC3806652

[ref41] Halgren T. A. The representation of van der Waals (vdW) interactions in molecular mechanics force fields: potential form, combination rules, and vdW parameters. J. Am. Chem. Soc. 1992, 114 (20), 7827–7843. 10.1021/ja00046a032.

[ref42] Sagui C.; Pedersen L. G.; Darden T. A. Towards an accurate representation of electrostatics in classical force fields: Efficient implementation of multipolar interactions in biomolecular simulations. J. Chem. Phys. 2004, 120 (1), 73–87. 10.1063/1.1630791. 15267263

[ref43] Schnieders M. J.; Fenn T. D.; Pande V. S. Polarizable atomic multipole X-ray refinement: Particle mesh Ewald electrostatics for macromolecular crystals. J. Chem. Theory Comput. 2011, 7 (4), 1141–1156. 10.1021/ct100506d. 26606362

[ref44] Wallace J. A.; Shen J. K. Continuous constant pH molecular dynamics in explicit solvent with pH-based replica exchange. J. Chem. Theory Comput. 2011, 7 (8), 2617–2629. 10.1021/ct200146j. 26606635 PMC6425487

[ref45] Groom C.; Bruno I.; Lightfoot M.; Ward S. Acta Crystallogr., Sect. B: Struct. Sci., Cryst. Eng. Mater. 2016, 72, 171 10.1107/S2052520616003954. PMC4822653 27048719

[ref46] Cheng F.; Sun H.; Zhang Y.; Mukkamala D.; Oldfield E. A solid state 13C NMR, crystallographic, and quantum chemical investigation of chemical shifts and hydrogen bonding in histidine dipeptides. J. Am. Chem. Soc. 2005, 127 (36), 12544–12554. 10.1021/ja051528c. 16144402

[ref47] Krause J. A.; Baures P.; Eggleston D. Molecular structures of l-Leu-l-Tyr, Gly-d, l-Met. p-toluenesulfonate and l-His-l-Leu. Acta Crystallographica Section B: Structural Science 1993, 49 (1), 123–130. 10.1107/S0108768192006207. 8442925

[ref48] Steiner T. L-Histidyl-L-alanine dihydrate. Acta Crystallographica Section C: Crystal Structure Communications 1996, 52 (10), 2554–2556. 10.1107/S0108270196006804.

[ref49] Mroué K. H.; Power W. P. High-Field Solid-State 67Zn NMR Spectroscopy of Several Zinc- Amino Acid Complexes. J. Phys. Chem. A 2010, 114 (1), 324–335. 10.1021/jp908325n. 19919076

[ref50] Williams P. A.; Hughes C. E.; Harris K. D. L-Lysine: Exploiting Powder X-ray Diffraction to Complete the Set of Crystal Structures of the 20 Directly Encoded Proteinogenic Amino Acids. Angew. Chem., Int. Ed. 2015, 54 (13), 3973–3977. 10.1002/anie.201411520. 25651303

[ref51] Rackers J. A.; Wang Z.; Lu C.; Laury M. L.; Lagardère L.; Schnieders M. J.; Piquemal J.-P.; Ren P.; Ponder J. W. Tinker 8: Software Tools for Molecular Design. J. Chem. Theory Comput. 2018, 14 (10), 5273–5289. 10.1021/acs.jctc.8b00529. 30176213 PMC6335969

[ref52] Donnini S.; Ullmann R. T.; Groenhof G.; Grubmüller H. Charge-neutral constant pH molecular dynamics simulations using a parsimonious proton buffer. J. Chem. Theory Comput. 2016, 12 (3), 1040–1051. 10.1021/acs.jctc.5b01160. 26881315

[ref53] Liu D. C.; Nocedal J. On the limited memory BFGS method for large scale optimization. Mathematical programming 1989, 45 (1–3), 503–528. 10.1007/BF01589116.

[ref54] Zhang C.; Lu C.; Jing Z.; Wu C.; Piquemal J.-P.; Ponder J. W.; Ren P. AMOEBA polarizable atomic multipole force field for nucleic acids. J. Chem. Theory Comput. 2018, 14 (4), 2084–2108. 10.1021/acs.jctc.7b01169. 29438622 PMC5893433

[ref55] Bennett C. H. Efficient estimation of free energy differences from Monte Carlo data. J. Comput. Phys. 1976, 22 (2), 245–268. 10.1016/0021-9991(76)90078-4.

[ref56] Eastman P.; Swails J.; Chodera J. D.; McGibbon R. T.; Zhao Y.; Beauchamp K. A.; Wang L.-P.; Simmonett A. C.; Harrigan M. P.; Stern C. D. OpenMM 7: Rapid development of high performance algorithms for molecular dynamics. PLoS computational biology 2017, 13 (7), e1005659 10.1371/journal.pcbi.1005659. 28746339 PMC5549999

[ref57] APEX, B. APEX4, SAINT, and SADABS; Bruker AXS Inc.: Madison, WI, 2021.

[ref58] Sheldrick G. M. SHELXT-Integrated space-group and crystal-structure determination. Acta Crystallographica Section A: Foundations and Advances 2015, 71 (1), 3–8. 10.1107/S2053273314026370. 25537383 PMC4283466

[ref59] Sheldrick G. M. Crystal structure refinement with SHELXL. Acta Crystallographica Section C: Structural Chemistry 2015, 71 (1), 3–8. 10.1107/S2053229614024218. 25567568 PMC4294323

[ref60] Dolomanov O. V.; Bourhis L. J.; Gildea R. J.; Howard J. A.; Puschmann H. OLEX2: a complete structure solution, refinement and analysis program. Journal of applied crystallography 2009, 42 (2), 339–341. 10.1107/S0021889808042726.

[ref61] Nessler A. J.; Okada O.; Hermon M. J.; Nagata H.; Schnieders M. J. Progressive alignment of crystals: reproducible and efficient assessment of crystal structure similarity. Journal of applied crystallography 2022, 55 (6), 1528–1537. 10.1107/S1600576722009670. 36570662 PMC9721330

[ref62] Ding X.; Vilseck J. Z.; Hayes R. L.; Brooks III C. L. Gibbs sampler-based λ-dynamics and Rao-Blackwell estimator for alchemical free energy calculation. J. Chem. Theory Comput. 2017, 13 (6), 2501–2510. 10.1021/acs.jctc.7b00204. 28510433 PMC5828534

[ref63] Corrigan R. A.; Thiel A. C.; Lynn J. R.; Casavant T. L.; Ren P.; Ponder J. W.; Schnieders M. J. A generalized Kirkwood implicit solvent for the polarizable AMOEBA protein model. J. Chem. Phys. 2023, 159 (5), 054102 10.1063/5.0158914. 37526158 PMC10396400

[ref64] Schnieders M. J.; Ponder J. W. Polarizable atomic multipole solutes in a generalized Kirkwood continuum. J. Chem. Theory Comput. 2007, 3 (6), 2083–2097. 10.1021/ct7001336. 26636202 PMC4767294

