# Supplementary material for: Constant-pH Simulations with the Polarizable Atomic Multipole AMOEBA Force Field
Source: J Chem Theory Comput. 2024 Mar 20;20(7):2921–33. doi: 10.1021/acs.jctc.3c01180 (PMC11008096; doi:10.1021/acs.jctc.3c01180)
Supplement: Supplementary file 1 — ct3c01180_si_001.pdf [file ct3c01180_si_001.pdf]

Supplemental Information for:

Constant-pH Simulations with the Polarizable Atomic Multipole AMOEBA Force Field

Andrew C. Thiel[1], Matthew J. Speranza [1], Sanika Jadhav[3], Lewis L. Stevens[3], Daniel K. Unruh[4], Pengyu Ren[5], Jay W. Ponder[6], Jana Shen[7], and Michael J. Schnieders*[1,2]

Departments of [1]Biomedical Engineering and [2]Biochemistry, University of Iowa 52242

Department of [3]Pharmaceutical Sciences and Experimental Therapeutics, University of Iowa 52242

[4]Office of the Vice President for Research, University of Iowa 52242

Department of [5]Biomedical Engineering, University of Texas, Austin, TX 78712

Department of [6]Chemistry, Washington University in St. Louis, St. Louis, MO 63130

Department of [7]Pharmaceutical Sciences, University of Maryland School of Pharmacy, Baltimore, MD 21201

*Corresponding Email: michael-schnieders@uiowa.edu

Table of Contents

Extended Terms 3

Extended Terms for Lysine and Cysteine 3

Extended Terms for Aspartic/Glutamic Acid 5

Extended Terms for Histidine 7

AMOEBA-2018-BIO CpHMD Parameter Modifications 10

Cysteine Parameter Changes: 10

Aspartate Parameter Changes: 11

Glutamate Parameter Changes: 13

Reversibility of the Mixed CPU/GPU CpHMD 14

Supplementary Figures 15

Figure S0. The titration curve of Lysine from 10 ns of the CPU-only implementation of CpHMD. This curve was generated to validate the usage of the CPU/GPU interleaving implementation reported in Figure 1 of the main text. The pKa reported for the interleaving approach was 10.35 which is in very close agreement with the pKa value of 10.32 measured for the CPU-only approach. 15

Figure S1 16

Extended Terms

Extended Terms for Lysine and Cysteine

Bias Terms.

Barrier Bias:

$$U_{barr} = 4\beta_t(\lambda_k - \frac{1}{2})^2$$

Barrier Bias Derivative:

$$\frac{\delta U_{barr}}{\delta \lambda} = 8\beta_t(\lambda_k - \frac{1}{2})$$

$$\beta_t = Bias\ Magnitude(\frac{Kcal}{mol})$$

pH Bias:

$$U_{pH} = \ln(10) k_\beta T(1 - \lambda_k)[(pK_\alpha^1 - pH)]$$

Lysine pK_α^1: 10.4

Cysteine pK_α^1: 8.55

pH Bias Derivative:

$$\frac{\delta U_{pH}}{\delta \lambda} = -\ln(10) k_\beta T[(pK_\alpha^1 - pH)]$$

$$k_\beta = \text{Boltzmann Constant}(\frac{kcal}{mol * K}), T = \text{temperature}(K)$$

$$pK_\alpha^1 = \text{Lysine acid dissocation constant for side chain}$$

Model Bias:

$$U_{mod} = A{\lambda_k}^3 + B{\lambda_k}^2 + C\lambda_k$$

Model Bias Derivative:

$$\frac{U_{mod}}{\delta \lambda} = 3A{\lambda_k}^2 + 2B\lambda_k + C$$

$$A, B, C = Fit\ parameters\ from\ BAR\ simulations$$

Lysine $A = 6.752, \mathrm{B} = -78.804, \mathrm{C} = 26.894$

Cysteine $A = 44.247, \mathrm{B} = -183.990, \mathrm{C} = 226.710$

Extended *van der Waals* Terms.

$$\tilde{U}_{vdw}(i,j) = \begin{cases} \lambda_i U_{vdw}(i,j) & j{:}other \\ \lambda_i \lambda_j U_{vdw}(i,j) & j{:}titrating\ K \\ \lambda_i \lambda_j f^m(\zeta_j) U_{vdw}(i,j) & j{:}titrating\ D,E \\ \lambda_i((1-\lambda_j) f^m(\zeta_j) + \lambda_j) U_{vdw}(i,j) & j{:}titrating\ H \end{cases}$$

$$f^m(\zeta_i) = \begin{cases} \zeta_i & m = 1 \\ 1 - \zeta_i & m = -1 \end{cases}$$

Extended Electrostatic Terms.

Interpolated Multipole:

$$\mathbf{M}_i(\lambda_k) = (1-\lambda_k)\mathbf{M}_i^{(\mathrm{U})} + \lambda_k \mathbf{M}_i^{(\mathrm{P})}$$

Interpolated Multipole Derivative:

$$\frac{\partial \mathbf{M}_i}{\partial \lambda_k} = \dot{\mathbf{M}}_i = \mathbf{M}_i^{(\mathrm{P})} - \mathbf{M}_i^{(\mathrm{U})}$$

Interpolated Polarizability:

$$\boldsymbol{\alpha}_i(\lambda_k) = (1-\lambda_k)\boldsymbol{\alpha}_i^{(\mathrm{U})} + \lambda_k \boldsymbol{\alpha}_i^{(\mathrm{P})}$$

Interpolated Polarizability Derivative:

$$\frac{\partial \boldsymbol{\alpha}_i}{\partial \lambda_k} = \boldsymbol{\alpha}_i^{(\mathrm{P})} - \boldsymbol{\alpha}_i^{(\mathrm{U})}$$

Extended Terms for Aspartic/Glutamic Acid

Bias Terms.

Barrier Bias:

$$U_{barr} = 4\beta_t(\lambda_k - \frac{1}{2})^2$$

$$U_{barr} = 4\beta_t(\zeta_k - \frac{1}{2})^2$$

Barrier Bias Derivative:

$$\frac{\delta U_{barr}}{\delta \lambda} = 8\beta_t(\lambda_k - \frac{1}{2})$$

$$\frac{\delta U_{barr}}{\delta \zeta} = 8\beta_t(\zeta_k - \frac{1}{2})$$

$$\beta_t = Bias\ Magnitude(\frac{Kcal}{mol})$$

pH Bias:

$$U_{pH} = \ln(10)k_\beta T(1-\lambda_k)[\zeta_k(pK_\alpha^1 - pH) + (1-\zeta_k)(pK_\alpha^2 - pH)]$$

pH Bias Derivative:

$$\frac{\delta U_{pH}}{\delta \lambda} = -\ln(10)k_\beta T[\zeta_k(pK_\alpha^1 - pH) + (1-\zeta_k)(pK_\alpha^2 - pH)]$$

$$\frac{\delta U_{pH}}{\delta \zeta} = \ln(10)k_\beta T(1-\lambda_k)[\ (pK_\alpha^1 - pH) - (pK_\alpha^2 - pH)]$$

$$k_\beta = Boltzmann\ Constant(\frac{kcal}{mol * K}), T = temperature(K)$$

$$pK_\alpha^1\ for\ side\ chain\ HD1/HE1\ ;\ pK_\alpha^2\ for\ side\ chain\ HD2/HE2$$

$$pK_\alpha^1 = pK_\alpha^2$$

Aspartic Acid pK_{α}^{1}: 3.94

Glutamic Acid pK_{α}^{1}: 4.25

2D Model Bias:

$$U_{mod} = \lambda_k * \left(a_0{\zeta_k}^3 + a_1{\zeta_k}^2 + a_2\zeta_k\right) + \zeta_k\left(b_0{\lambda_k}^3 + b_1{\lambda_k}^2 + b_2\lambda_k\right) + (1-\zeta_k) * \left(c_0{\lambda_k}^3 + c_1{\lambda_k}^2 + c_2\lambda_k\right) + (1-\lambda_k)\zeta_k(C-B)$$

2D Model Bias Derivative:

$$\frac{U^{mod}}{\delta\lambda} = \left(a_0{\zeta_k}^3 + a_1{\zeta_k}^2 + a_2\zeta_k\right) + \zeta_k\left(3b_0{\lambda_k}^2 + 2b_1\lambda_k + b_2\right) + (1-\zeta_k)\left(3c_0{\lambda_k}^2 + 2c_1\lambda_k + c_2\right) - \zeta_k(C-B)$$

$$\frac{U^{mod}}{\delta\zeta} = \lambda_k * \left(3a_0{\zeta_k}^2 + 2a_1\zeta_k + a_2\right) + \left(b_0{\lambda_k}^3 + b_1{\lambda_k}^2 + b_2\lambda_k\right) - \left(c_0{\lambda_k}^3 + c_1{\lambda_k}^2 + c_2\lambda_k\right) + (1-\lambda_k)(C-B)$$

$$a_0, b_0, c_0, etc. = Fit\ parameters\ from\ BAR\ simulations$$

ASH2_ASH1: $a_0 = 0.0, a_1 = -35.505,\ a_2 = 35.505$

ASP_ASH: $b_0, c_0 = 12.730, b_1, c_1 = -107.430,\ b_2, c_2 = 166.369$

GLH2_GLH1: $a_0 = 0.0, a_1 = -29.395,\ a_2 = 29.395$

GLU_GLH: $b_0, c_0 = 28.024, b_1, c_1 = -131.27,\ b_2, c_2 = 189.98$

$$C = c_0 + c_1 + c_2;\ B = b_0 + b_1 + b_2$$

Extended *van der Waals* Terms.

$$\tilde{U}_{vdw}(i,j) = \begin{cases} \lambda_i f^m(\zeta_i) U_{vdw}(i,j) & j{:}other \\ \lambda_i f^m(\zeta_i)\lambda_j U_{vdw}(i,j) & j{:}titrating\ K \\ \lambda_i f^m(\zeta_i)\lambda_j f^m(\zeta_j) U_{vdw}(i,j) & j{:}titrating\ D,E \\ \lambda_i f^m(\zeta_i)\left((1-\lambda_j) f^m(\zeta_j) + \lambda_j\right) U_{vdw}(i,j) & j{:}titrating\ H \end{cases}$$

$$f^m(x_i) = \begin{cases} \zeta_i & m = 1 \\ 1-\zeta_i & m = -1 \end{cases}$$

Extended Electrostatic Terms.

Interpolated Multipole:

$$\mathbf{M}_i(\lambda_k,\zeta_k) = (1-\lambda_k)\mathbf{M}_i^{(\mathrm{U})} + \lambda_k(\zeta_k\mathbf{M}_i^{(\mathrm{P1})} + (\mathbf{1}-\zeta_k)\mathbf{M}_i^{(\mathrm{P2})})$$

Interpolated Multipole Derivative:

$$\frac{\partial \mathbf{M}_i}{\partial \lambda_k} = \dot{\mathbf{M}}_i = (\zeta_k\mathbf{M}_i^{(\mathrm{P1})} + (\mathbf{1}-\zeta_k)\mathbf{M}_i^{(\mathrm{P2})}) - \mathbf{M}_i^{(\mathrm{U})}$$

$$\frac{\partial \mathbf{M}_i}{\partial \zeta_k} = \lambda_k(\mathbf{M}_i^{(\mathrm{P1})} - \mathbf{M}_i^{(\mathrm{P2})})$$

Interpolated Polarizability:

$$\boldsymbol{\alpha}_i(\lambda_k,\zeta_k) = (1-\lambda_k)\boldsymbol{\alpha}_i^{(\mathrm{U})} + \lambda_k(\zeta_k\boldsymbol{\alpha}_i^{(\mathrm{P1})} + (\mathbf{1}-\zeta_k)\boldsymbol{\alpha}_i^{(\mathrm{P2})})$$

Interpolated Polarizability Derivative:

$$\frac{\partial \boldsymbol{\alpha}_i}{\partial \lambda_k} = (\zeta_k\boldsymbol{\alpha}_i^{(\mathrm{P1})} + (\mathbf{1}-\zeta_k)\boldsymbol{\alpha}_i^{(\mathrm{P2})}) - \boldsymbol{\alpha}_i^{(\mathrm{U})}$$

$$\frac{\partial \boldsymbol{\alpha}_i}{\partial \zeta_k} = \lambda_k(\boldsymbol{\alpha}_i^{(\mathrm{P1})} - \boldsymbol{\alpha}_i^{(\mathrm{P2})})$$

Extended Terms for Histidine

Bias Terms.

Barrier Bias:

$$U_{barr} = 4\beta_t(\lambda_k - \frac{1}{2})^2$$

$$U_{barr} = 4\beta_t(\zeta_k - \frac{1}{2})^2$$

Barrier Bias Derivative:

$$\frac{\delta U_{barr}}{\delta \lambda} = 8\beta_t(\lambda_k - \frac{1}{2})$$

$$\frac{\delta U_{barr}}{\delta \zeta} = 8\beta_t(\zeta_k - \frac{1}{2})$$

$$\beta_t = Bias\ Magnitude(\frac{Kcal}{mol})$$

pH Bias:

$$U_{pH} = \ln(10) k_\beta T (1 - \lambda_k)[\zeta_k(pK_\alpha^1 - pH) + (1 - \zeta_k)(pK_\alpha^2 - pH)]$$

pH Bias Derivative:

$$\frac{\delta U_{pH}}{\delta \lambda} = -\ln(10) k_\beta T[\zeta_k(pK_\alpha^1 - pH) + (1 - \zeta_k)(pK_\alpha^2 - pH)]$$

$$\frac{\delta U_{pH}}{\delta \zeta} = \ln(10) k_\beta T (1 - \lambda_k)[\ (pK_\alpha^1 - pH) - (pK_\alpha^2 - pH)]$$

$$k_\beta = Boltzmann\ Constant(\frac{kcal}{mol * K}), T = temperature(K)$$

$$pK_\alpha^1 = 6.60\ for\ side\ chain\ HD1$$

$$pK_\alpha^2 = 7.00\ for\ side\ chain\ HE2$$

2D Model Bias:

$$U_{mod} = (1 - \lambda_k) * (a_0 {\zeta_k}^3 + a_1 {\zeta_k}^2 + a_2 \zeta_k) + \zeta_k(b_0 {\lambda_k}^3 + b_1 {\lambda_k}^2 + b_2 \lambda_k) + (1 - \zeta_k) * (c_0 {\lambda_k}^3 + c_1 {\lambda_k}^2 + c_2 \lambda_k) + \lambda_k \zeta_k (C - B)$$

2D Model Bias Derivative:

$$\frac{U^{mod}}{\delta \lambda} = -(a_0 {\zeta_k}^3 + a_1 {\zeta_k}^2 + a_2 \zeta_k) + \zeta_k(3b_0 {\lambda_k}^2 + 2b_1 \lambda_k + b_2) + (1 - \zeta_k)(3c_0 {\lambda_k}^2 + 2c_1 \lambda_k + c_2) + \zeta_k(C - B)$$

$$\frac{U^{mod}}{\delta \zeta} = (1 - \lambda_k) * (3a_0 {\zeta_k}^2 + 2a_1 \zeta_k + a_2) + (b_0 {\lambda_k}^3 + b_1 {\lambda_k}^2 + b_2 \lambda_k) - (c_0 {\lambda_k}^3 + c_1 {\lambda_k}^2 + c_2 \lambda_k) + \lambda_k (C - B)$$

$$a_0, b_0, c_0, etc. = Fit\ parameters\ from\ BAR\ simulations$$

$$\text{HID\_HIE: } a_0 = 0.0, a_1 = -36.83, a_2 = 34.325$$

$$\text{HIE\_HIS: } b_0 = 0.0, b_1 = -62.931, b_2 = 32.00$$

$$\text{HID\_HIS: } c_0 = 0.0, c_1 = -64.317, b_2 = 30.350$$

$$C = c_0 + c_1 + c_2; B = b_0 + b_1 + b_2$$

Extended *van der Waals* Terms.

$$\tilde{U}_{vdw}(i,j) = \begin{cases} ((1-\lambda_i)f^m(\zeta_i) + \lambda_i)U_{vdw}(i,j) & j{:}other \\ ((1-\lambda_i)f^m(\zeta_i) + \lambda_i)\lambda_j U_{vdw}(i,j) & j{:}titrating\ K \\ ((1-\lambda_i)f^m(\zeta_i) + \lambda_i)\lambda_j f^m(\zeta_j)U_{vdw}(i,j) & j{:}titrating\ D,E \\ ((1-\lambda_i)f^m(\zeta_i) + \lambda_i)((1-\lambda_j)f^m(\zeta_j) + \lambda_j)U_{vdw}(i,j) & j{:}titrating\ H \end{cases}$$

$$f^m(x_i) = \begin{cases} \zeta_i & m = 1 \\ 1-\zeta_i & m = -1 \end{cases}$$

Extended Electrostatic Terms.

Interpolated Multipole:

$$\mathbf{M}_i(\lambda_k,\zeta_k) = \lambda_k \mathbf{M}_i^{(\mathrm{HIS})} + (\mathbf{1}-\lambda_k)(\zeta_k \mathbf{M}_i^{(\mathrm{HIE})} + (\mathbf{1}-\zeta_k)\mathbf{M}_i^{(\mathrm{HID})})$$

Interpolated Multipole Derivative:

$$\frac{\partial \mathbf{M}_i}{\partial \lambda_k} = \dot{\mathbf{M}}_i = \mathbf{M}_i^{(\mathrm{HIS})} - (\zeta_k \mathbf{M}_i^{(\mathrm{HIE})} + (\mathbf{1}-\zeta_k)\mathbf{M}_i^{(\mathrm{HID})})$$

$$\frac{\partial \mathbf{M}_i}{\partial \zeta_k} = (\mathbf{1}-\lambda_k)(\mathbf{M}_i^{(\mathrm{HIE})} - \mathbf{M}_i^{(\mathrm{HID})})$$

Interpolated Polarizability:

$$\boldsymbol{\alpha}_i(\lambda_k,\zeta_k) = \lambda_k \boldsymbol{\alpha}_i^{(\mathrm{HIS})} + (\mathbf{1}-\lambda_k)(\zeta_k \boldsymbol{\alpha}_i^{(\mathrm{HIE})} + (\mathbf{1}-\zeta_k)\boldsymbol{\alpha}_i^{(\mathrm{HID})})$$

Interpolated Polarizability Derivative:

$$\frac{\partial \boldsymbol{\alpha}_i}{\partial \lambda_k} = \boldsymbol{\alpha}_i^{(\mathrm{HIS})} - (\zeta_k \boldsymbol{\alpha}_i^{(\mathrm{HIE})} + (\mathbf{1}-\zeta_k)\boldsymbol{\alpha}_i^{(\mathrm{HID})})$$

$$\frac{\partial \boldsymbol{\alpha}_i}{\partial \zeta_k} = (\mathbf{1}-\lambda_k)(\boldsymbol{\alpha}_i^{(\mathrm{HIE})} - \boldsymbol{\alpha}_i^{(\mathrm{HID})})$$

AMOEBA-2018-BIO CpHMD Parameter Modifications

Cysteine Parameter Changes:

Old:

```
#biotype    97  CA    "Cysteine (S-)          "     48
```

New:

```
biotype    97  CA    "Cysteine (S-)          "      8
```

Old:

```
#biotype   458  CA    "N-Terminal CYD (S-)    "     48
```

New:

```
biotype    639  CA    "C-Terminal CYD (S-)    "      8
```

Old:

```
#multipole    43     48     49         -0.08027 \
#                             0.13313  0.00000  0.28094 \
#                            -0.73267 \
#                             0.00000  0.44660 \
#                            -0.05681  0.00000  0.28607
```

New:

Note: (Changed the partial charge from (q = -0.08027) by (-0.293) to below to maintain charge neutrality with new HB multipole definition.)

```
multipole     43     8     49                  -0.373272 \
                0.13313  0.00000  0.28094 \
               -0.73267 \
                0.00000  0.44660 \
               -0.05681  0.00000  0.28607
```

Old:

```
# multipole    45    46    43       -0.03264 \
#                          0.49460  0.00000 -0.04347 \
#                          1.63727 \
#                          0.00000 -2.40778 \
#                         -0.45566  0.00000  0.77051
```

New:

Note: (CYS SG cannot use the HG atom for its frame during CpHMD
The new SG frame uses CB then CA (as does CYD SG); the multipole was updated for the new frame.)

```
multipole    45   43    8            -0.03264 \
                          -0.00847    0.00000    0.49643 \
                           0.68524 \
                           0.00000   -2.40778 \
                           0.35560    0.00000    1.72254
```

Old:

```
# multipole    49    43    48       -0.85155 \
#                          0.15871  0.00000  0.56464 \
#                         -1.49210 \
#                          0.00000 -1.58110 \
#                         -0.20334  0.00000  3.07320
```

New:

Note: (CYD SG- now uses the default CA multipole (48 replaced with 8 below); the components do not need to be changed.)

```
multipole     49     43     8         -0.85155 \
                          0.15871  0.00000  0.56464 \
                         -1.49210 \
                          0.00000 -1.58110 \
                         -0.20334  0.00000  3.07320
```

Old:

```
# polarize     43   1.33400  0.39000     44     48     49
```

New:

```
polarize     43   1.33400  0.39000     44     45     49
```

Old:

```
# polarize     45   2.80000  0.39000     46
```

New:

```
polarize     45   2.80000  0.39000     43    46
```

Note: (For protonated CYS, SG and HG were originally their own polarization group.
Here we expand the polarization group to include CB (43) to match the CYD.
In principle, the SG multipole should be re-optimized to account for the change in polarization group.
The polarization group list removed 48 which was a special type of the CA for CYD
As of October, 2023 this has not been done.)

Unused Parameters:

```
# multipole    48     7     9        -0.23113 \
#                          0.27663  0.00000  0.12440 \
#                         -0.20625 \
#                          0.00000 -0.28389 \
#                          0.15574  0.00000  0.49014
# multipole    48     7   233          0.60118 \
#                          0.27663  0.00000  0.12440 \
```

```
#                              -0.20625 \
#                               0.00000 -0.28389 \
#                               0.15574  0.00000  0.49014
```

Aspartate Parameter Changes:

Old:

```
# multipole   121    120    124        0.13110 \
#                               0.02575  0.00000 -0.14558 \
#                               0.00657 \
#                               0.00000 -0.01572 \
#                               0.00411  0.00000  0.00915
```

New:

```
multipole   121  120  119              0.13110 \
                                -0.02575    0.00000   -0.14558 \
                                 0.00657 \
                                 0.00000   -0.01572 \
                                -0.00411    0.00000    0.00915
```

Old:

```
# Replaced by values for CpHMD at the end of the file.
# multipole   139    140   -140        1.01811 \
#                              -0.00488  0.00000 -0.15412 \
#                               0.00000 \
#                               0.00000  0.00000 \
#                               0.00000  0.00000  0.00000
```

New:

Note:(replaced AMOEBA-BIO-18 multipole that has a non-zero x-dipole)

```
multipole   139  -140 -140             1.01811 \
                                 0.00000    0.00000   -0.15412 \
                                 0.00000 \
                                 0.00000    0.00000 \
                                 0.00000    0.00000    0.00000
```

Old:

```
# multipole   140    139    137       -0.85879 \
#                              -0.08949  0.00000 -0.07764 \
#                               0.00000 \
#                               0.00000  0.00000 \
#                               0.00000  0.00000  0.00000
```

New:

```
multipole   140  139  140             -0.85879 \
                                 0.08949    0.00000   -0.07764 \
                                 0.00000 \
                                 0.00000    0.00000 \
                                 0.00000    0.00000    0.00000
```

Old:

```
# multipole   143    144    145        0.85923 \
#                               0.11304  0.00000  0.10040 \
#                               0.31104 \
#                               0.00000 -0.30726 \
#                              -0.25489  0.00000 -0.00378
```

New:

```
Note: (replaced 143 144 145 Z-then-X with Bisector)
multipole   143 -144 -145              0.85923 \
                                  0.00000    0.00000    0.14630 \
                                  0.26732 \
                                  0.00000   -0.30726 \
                                  0.00000    0.00000    0.03994
```

Old:

```
# multipole    145    143    146        -0.43939 \
#                        0.21541  0.00000 -0.02221 \
#                        0.36981 \
#                        0.00000 -0.36139 \
#                       -0.26932  0.00000 -0.00842
```

New:

```
multipole   145  143  144             -0.43939 \
                                  0.21541    0.00000   -0.02221 \
                                  0.36981 \
                                  0.00000   -0.36139 \
                                 -0.26932    0.00000   -0.00842
```

Glutamate Parameter Changes:

Old:

```
# multipole    157    158   -158         1.05691 \
#                       -0.08698  0.00000  0.00867 \
#                        0.00000 \
#                        0.00000  0.00000 \
#                        0.00000  0.00000  0.00000
```

New:

```
Note: (replaced AMOEBA-BIO-18 which has a non-zero x-dipole)
multipole   157 -158 -158              1.05691 \
                                  0.00000    0.00000    0.00867 \
                                  0.00000 \
                                  0.00000    0.00000 \
                                  0.00000    0.00000    0.00000
```

Old:

```
# multipole    160    159      8         0.09513 \
#                       -0.05887  0.00000 -0.04640 \
#                       -0.03611 \
#                        0.00000 -0.02016 \
#                       -0.01670  0.00000  0.05627
```

New:

```
multipole   160  159  161              0.09513 \
                                  0.02939    0.05101   -0.04640 \
                                 -0.02414 \
                                 -0.00690   -0.03213 \
                                  0.00834    0.01447    0.05627
```

Old:

```
# multipole    163    164    165         0.90086 \
#                        0.11874  0.00000  0.10181 \
#                       -0.08663 \
```

```
#                                0.00000  0.03649 \
#                               -0.12175  0.00000  0.05014
```

New:

Note: (replaced 163 164 165 Z-then-X with Bisector)

```
multipole   163 -164 -165              0.90086 \
                                 0.00000    0.00000    0.15202 \
                                 0.12045 \
                                 0.00000    0.03649 \
                                 0.00000    0.00000   -0.15694
```

Reversibility of the Mixed CPU/GPU CpHMD

To illustrate microscopic reversibility of the mixed CPU/GPU CpHMD, first simulations in the microcanonical ensemble (NVE) will be described, followed by introduction of a thermostat to sample from the canonical ensemble (NVT). Consider a simulation in the NVE ensemble using a time-reversible area-preserving integrator such as velocity Verlet. The total Hamiltonian on the CPU at time step *t* is given by

$$H^{CPU}(\mathbf{X}_t, \dot{\mathbf{X}}_t, \boldsymbol{\lambda}_t, \dot{\boldsymbol{\lambda}}_t) = E(\mathbf{X}_t, \boldsymbol{\lambda}_t) + E^{\mathrm{Titr}}(\boldsymbol{\lambda}_t) + K(\dot{\mathbf{X}}_t) + K(\dot{\boldsymbol{\lambda}}_t)$$

Equation 1.

where $\mathbf{X}_t$ are atomic coordinates, $\boldsymbol{\lambda}_t$ are extended system titration/tautomer coordinates, *E* is the AMOEBA potential energy as evaluated on either the CPU or GPU, E^{Titr} are titration / tautomer dependent titration energy terms (evaluated only on the CPU), and *K* is the kinetic energy, which is a function of atomic coordinate velocities $\dot{\mathbf{X}}_t$ and extended system titration/tautomer velocities $\dot{\boldsymbol{\lambda}}_t$.

The initial total energy is given by $H^{CPU}(\mathbf{X}_0, \dot{\mathbf{X}}_0, \boldsymbol{\lambda}_0, \dot{\boldsymbol{\lambda}}_0)$ and at an arbitrary time in the future denoted by step *1* is given by $H^{CPU}(\mathbf{X}_1, \dot{\mathbf{X}}_1, \boldsymbol{\lambda}_1, \dot{\boldsymbol{\lambda}}_1)$ where the total energy has been conserved (clearly potential and kinetic energy can exchange). We then have:

$$H^{CPU}(\mathbf{X}_0, \dot{\mathbf{X}}_0, \boldsymbol{\lambda}_0, \dot{\boldsymbol{\lambda}}_0) = H^{CPU}(\mathbf{X}_1, \dot{\mathbf{X}}_1, \boldsymbol{\lambda}_1, \dot{\boldsymbol{\lambda}}_1) = E(\mathbf{X}_1, \boldsymbol{\lambda}_1) + E^{\mathrm{Titr}}(\boldsymbol{\lambda}_1) + K(\dot{\mathbf{X}}_1) + K(\dot{\boldsymbol{\lambda}}_1)$$

Equation 2.

We now continue with NVE dynamics using velocity Verlet on the GPU using the Hamiltonian

$$H^{GPU}(\mathbf{X}_t, \dot{\mathbf{X}}_t) = E(\mathbf{X}_t, \boldsymbol{\lambda}_t = \hat{\boldsymbol{\lambda}}) + K(\dot{\mathbf{X}}_t)$$

Equation 3.

where $\boldsymbol{\lambda}_t = \hat{\boldsymbol{\lambda}}$ denotes that at any time *t* the extended degrees of freedom have been held constant. Thus, the initial GPU Hamiltonian at step 1 is given by (*i.e.*, continuing from the CPU values from step 1: $\mathbf{X}_1, \dot{\mathbf{X}}_1, \boldsymbol{\lambda}_1, \dot{\boldsymbol{\lambda}}_1$).

$$H^{GPU}(\mathbf{X}_1, \dot{\mathbf{X}}_1) = E(\mathbf{X}_1, \boldsymbol{\lambda}_1) + K(\dot{\mathbf{X}}_1)$$

Equation 4.

The final energy at some arbitrary time in the future when the GPU move is terminated is denoted as step 2 and given by

$$H^{GPU}(\mathbf{X}_1, \dot{\mathbf{X}}_1) = H^{GPU}(\mathbf{X}_2, \dot{\mathbf{X}}_2) = E(\mathbf{X}_2, \boldsymbol{\lambda}_1) + K(\dot{\mathbf{X}}_2)$$

Equation 5.

where the extended system variables have remained constant $\boldsymbol{\lambda}_2 = \boldsymbol{\lambda}_1$. Based on Eq. 5, the final (step 2) GPU Hamiltonian is substituted back into the overall CPU Hamiltonian to yield:

$$H^{CPU}(\mathbf{X}_0, \dot{\mathbf{X}}_0, \boldsymbol{\lambda}_0, \dot{\boldsymbol{\lambda}}_0) = H^{CPU}(\mathbf{X}_1, \dot{\mathbf{X}}_1, \boldsymbol{\lambda}_1, \dot{\boldsymbol{\lambda}}_1) = H^{CPU}(\mathbf{X}_2, \dot{\mathbf{X}}_2, \boldsymbol{\lambda}_1, \dot{\boldsymbol{\lambda}}_1) = E(\mathbf{X}_2, \boldsymbol{\lambda}_1) + E^{\mathrm{Titr}}(\boldsymbol{\lambda}_1) + K(\dot{\mathbf{X}}_2) + K(\dot{\boldsymbol{\lambda}}_1)$$

Equation 6.

The net result is that energy has been conserved while atomic coordinates have undergone two time-reversible moves and the extended system degrees of freedom one time-reversible move. The entire procedure can be executed in reverse beginning from the parameters $(\mathbf{X}_2, -\dot{\mathbf{X}}_2, \boldsymbol{\lambda}_1, -\dot{\boldsymbol{\lambda}}_1)$ where the signs of the velocities have been negated.

To sample from the NVT ensemble, the thermostat described by Bussi *et al.* (Equations 2 and 3 in that work[1]) can be applied at the beginning of each CPU move (*i.e.*, draw a target total kinetic energy from the canonical equilibrium distribution, which is then used to define a single velocity scale factor that is applied to all particle $\dot{\mathbf{X}}_t$ and extended system $\dot{\boldsymbol{\lambda}}_t$ velocities). After velocity scaling, the system evolves under Hamilton's equations as described above. Application of a Metropolis MC criteria when moving between the GPU and CPU under such NVT conditions is unnecessary due to energy conservation. Each move would be accepted because the change in energy is zero: $H^{CPU}(\mathbf{X}_1,\boldsymbol{\lambda}_1) - H^{CPU}(\mathbf{X}_2,\boldsymbol{\lambda}_1) = 0$.

Alternatively, sampling from the NVT ensemble can also proceed via stochastic dynamics, which was chosen in this work. Note that relaxation of this AMOEBA CpHMD model is not designed to provide meaningful kinetics (*i.e.*, diffusion of protons throughout the system is clearly not taken into account). As the OpenMM and Tinker GPU codes are modified to support Eq. 1 natively, this sampling strategy will become unnecessary.

(1) Bussi, G.; Donadio, D.; Parrinello, M. Canonical sampling through velocity rescaling. *The Journal of Chemical Physics* **2007**, *126* (1). DOI: 10.1063/1.2408420 (acccessed 1/4/2024).

Supplementary Figures

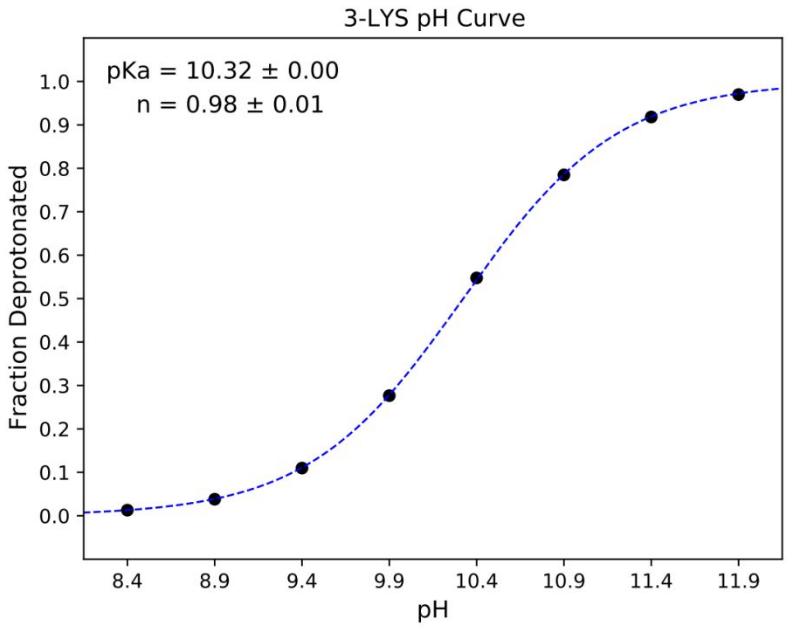

Figure S0. The titration curve of Lysine from 10 ns of the CPU-only implementation of CpHMD. This curve was generated to validate the usage of the CPU/GPU interleaving implementation reported in Figure 1 of the main text. The pKa reported for the interleaving approach was 10.35 which is in very close agreement with the pKa value of 10.32 measured for the CPU-only approach.

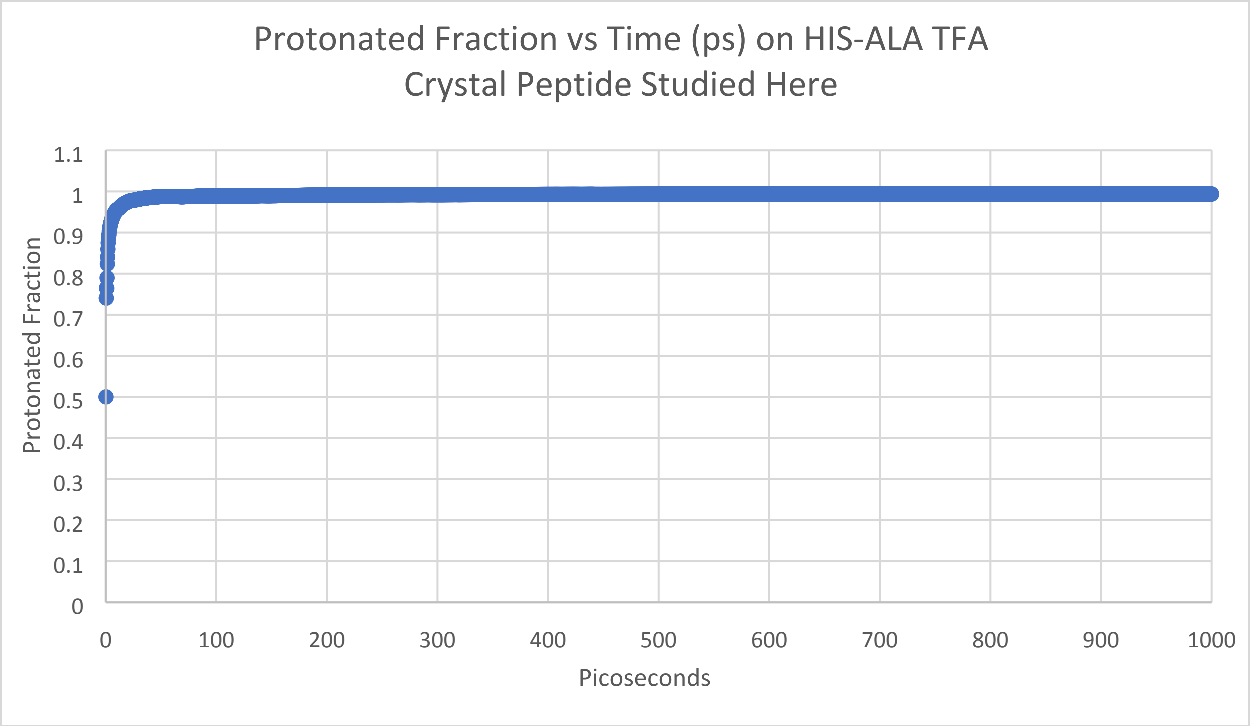

Figure S1. Time series for the HIS-ALA TFA studied here. Convergence of the protonated fraction occurs within the first 100 ps of the simulation, which remains stable beyond 10 ns of sampling (only 1 ns is shown here).
